# Supplementary material for: Modulation of p53 expression in cancer-associated fibroblasts prevents peritoneal metastasis of gastric cancer
Source: Mol Ther Oncolytics. 2022 Apr 25;25:249–61. doi: 10.1016/j.omto.2022.04.009 (PMC9108396; doi:10.1016/j.omto.2022.04.009)
Supplement: Document S1. Figures S1–S7 [file mmc1.pdf]

OMTO, Volume *25*

Supplemental information

Modulation of *p53* expression in cancer-associated fibroblasts prevents peritoneal metastasis of gastric cancer

Toshihiro Ogawa, Satoru Kikuchi, Motoyasu Tabuchi, Ema Mitsui, Yuta Une, Hiroshi Tazawa, Shinji Kuroda, Kazuhiro Noma, Toshiaki Ohara, Shunsuke Kagawa, Yasuo Urata, and Toshiyoshi Fujiwara

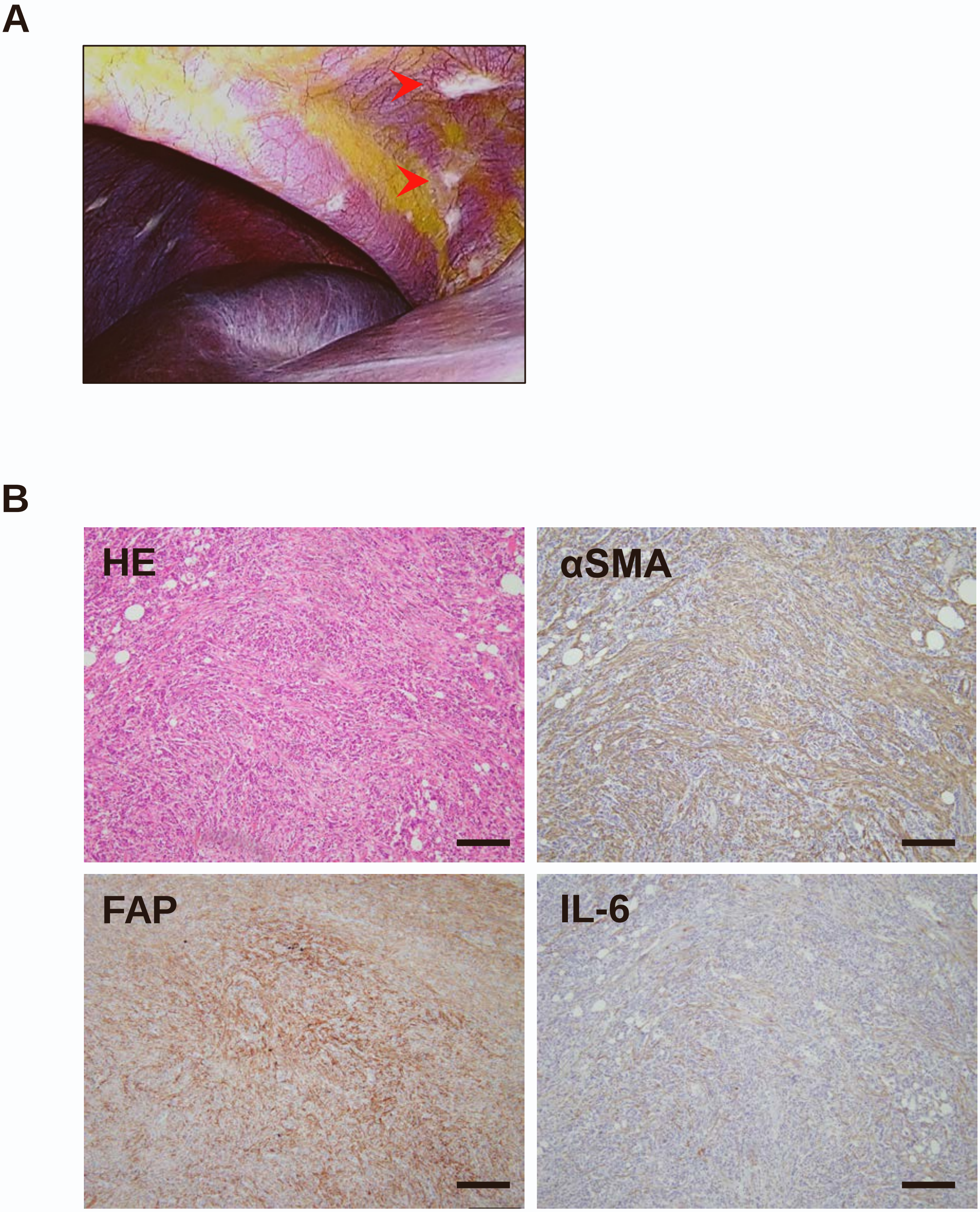

Supplementary Fig. S1

Immunohistochemical analysis of peritoneal metastasis of GC. **A,** Laparoscopic view of peritoneal metastasis of GC. White nodules indicates peritoneal metastasis (arrowheads). **B,** Representative microscopic images of peritoneal metastasis stained by HE and IHC staining for αSMA, FAP and IL-6. Scale bar, 200μm.

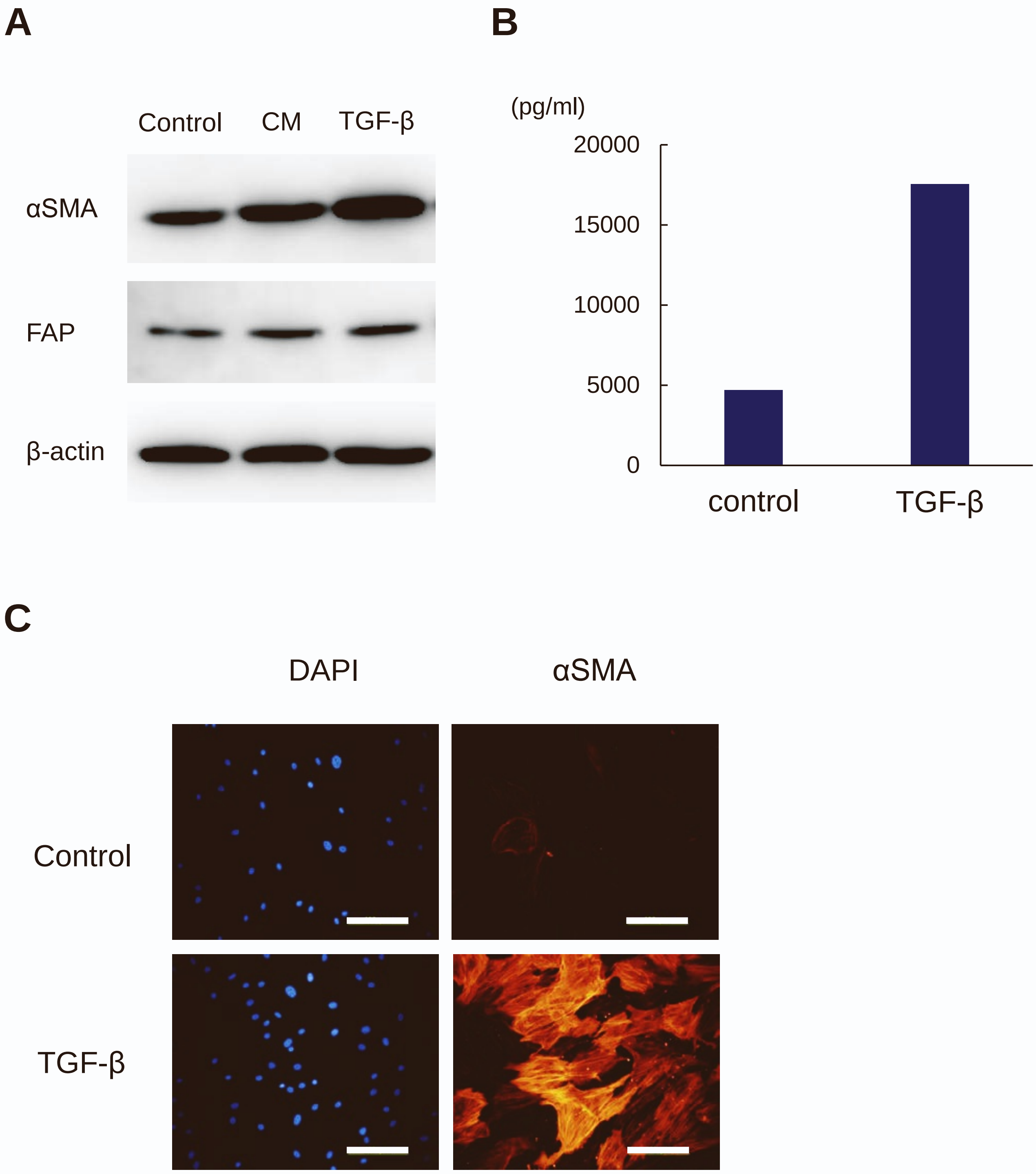

Supplementary Fig. S2

NGF activated by CM from MKN-7 or TGF-β showed CAFs-like properties. **A,** Western blotting analysis of NGF and activated NGF by CM from MKN-7 or TGF-β. β-actin was used as a loading control.. **B,** The amount of IL-6 secretion in NGF and activated NGF by TGF-β. **C,** Immunofluorescence analysis of NGF and activated NGF by TGF-β. Blue color indicates the nucleus, orange color indicates αSMA expression. Scale bar, 200μm.

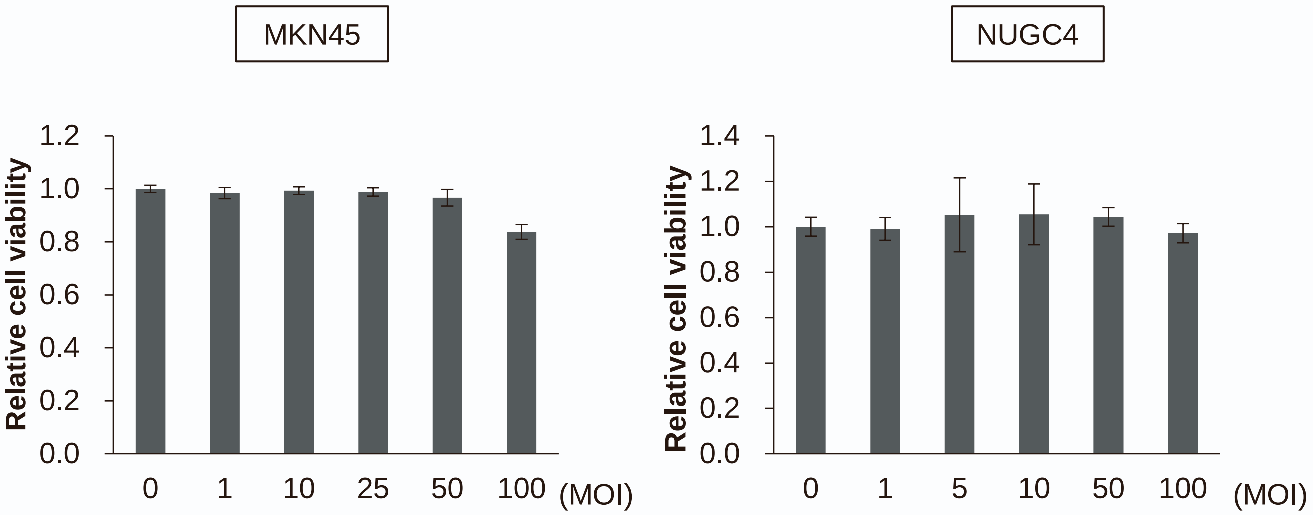

Supplementary Fig. S3

MKN45 and NUGC4 cells were resistant to OBP-301. Cells were infected with OBP-301 at the indicated MOIs for 3 days. Cell viability was quantified using the XTT assay. The cell viability of a mock-treated group was considered 1.0, and the relative cell viability was calculated. Data are expressed as the mean ± SD (n = 5).

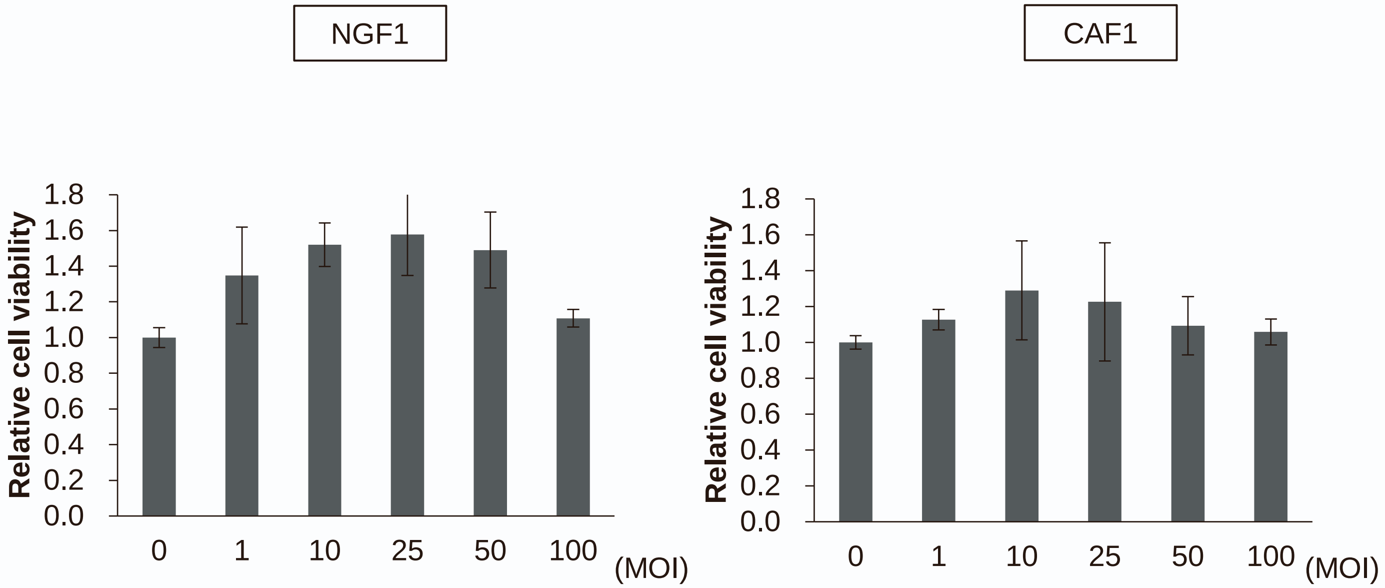

Supplementary FigS4

OBP-301 has no cytotoxicity to NGF and CAF. NGF1 and CAF1 were infected with OBP-301 at the indicated MOIs for 3 days. Cell viability was quantified using the XTT assay. The cell viability of a mock-treated group was considered 1.0, and the relative cell viability was calculated. Data are expressed as the mean ± SD (n = 5).

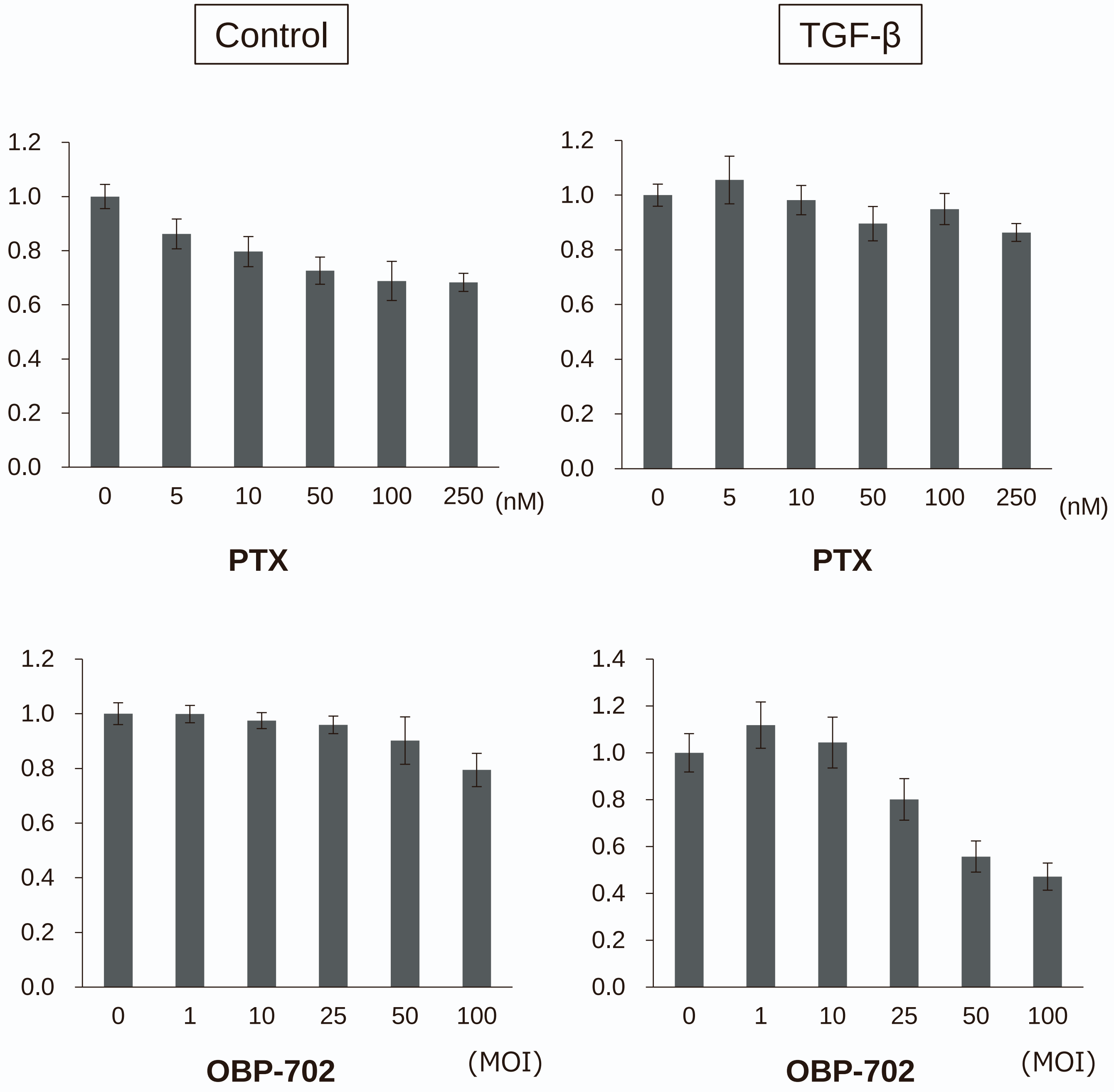

Supplementary FigS5

Activated NGF by TGF-β became to be OBP-702 sensitive and PTX resistant. NGF2 and activated NGF2 by TGF-β were infected with OBP-702 at the indicated MOIs for 3 days. Cells were treated with PTX at the indicated doses for 24 h. Cell viability was quantified using the XTT assay. The cell viability of a mock-treated group was considered 1.0, and the relative cell viability was calculated. Data are expressed as the mean ± SD (n = 5).

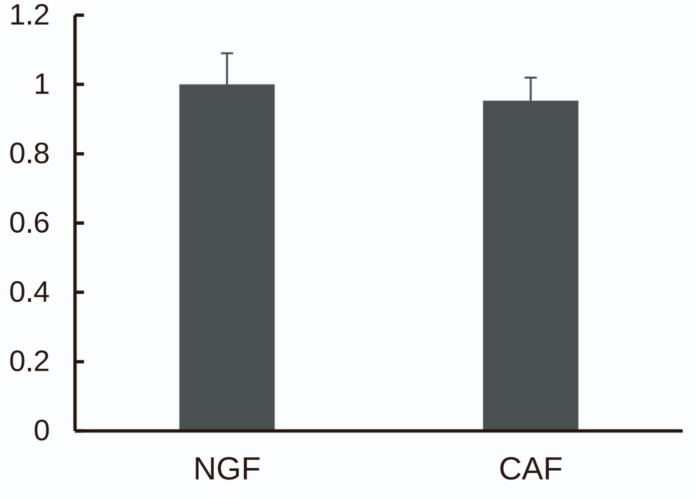

Supplementary FigS6
hTERT expression between NGF and CAF. Quantitative real-time PCR assay was performed to quantify the amount of hTERT copy. The copy number of hTERT is defined as the hTERT/ GAPDH ratio relative to that of the sample NGF (NGF = 1). Data are shown as means ± SD.

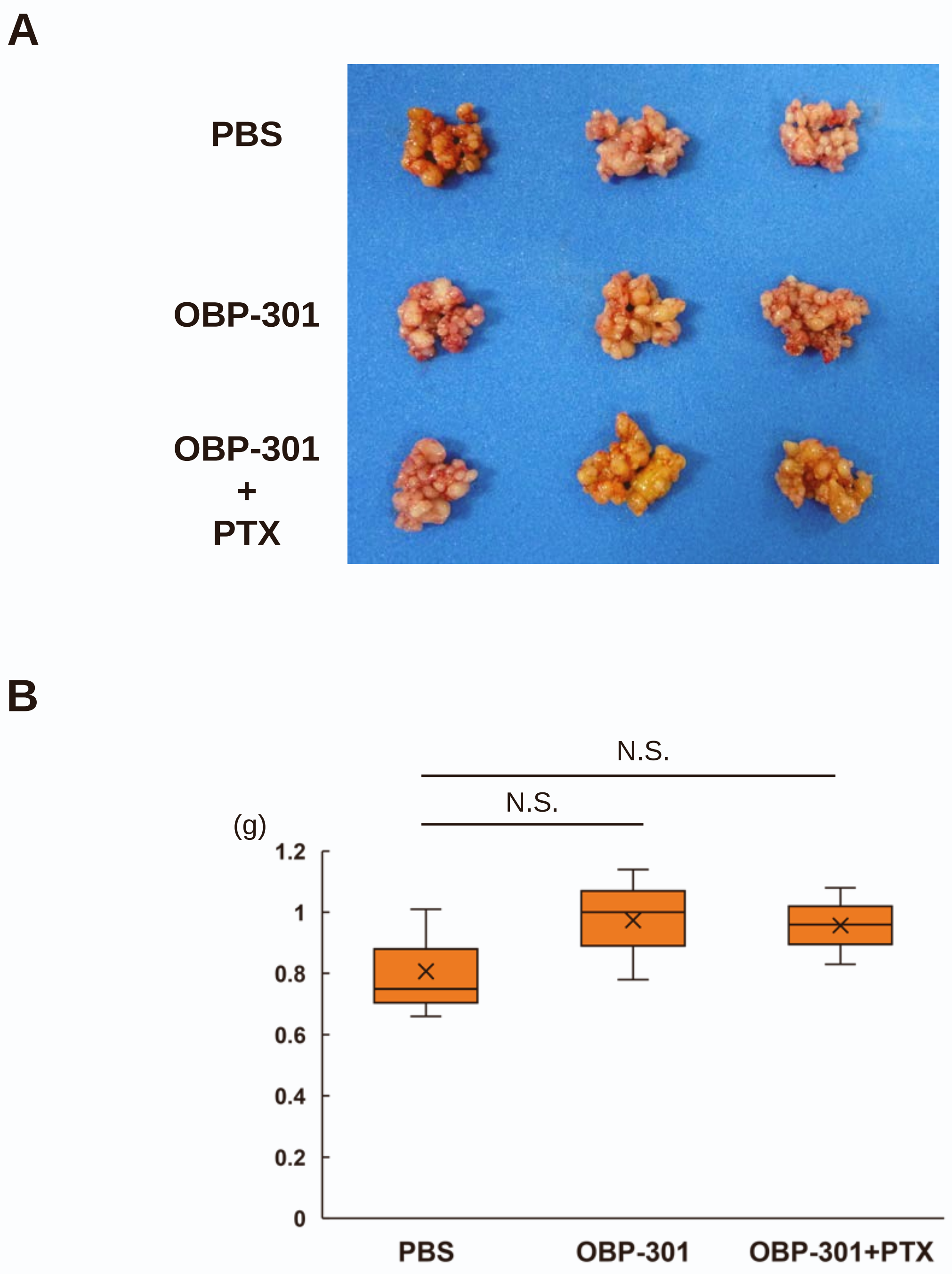

Supplementary FigS7
Intraperitoneal administration of OBP-301 in combination with PTX did not suppress tumor growth of MKN45-Luc and CAFs. **A,** Macroscopic images of resected all tumor nodules in abdominal cavity after treatment. B, Total tumor volume weight in abdominal cavity after treatment. Data are expressed as mean ± SD (n = 3). Statistical significance was defined as $p < 0.05$.
