# Supplementary material for: Modulation of p53 expression in cancer-associated fibroblasts prevents peritoneal metastasis of gastric cancer
Source: Mol Ther Oncolytics. 2022 Apr 25;25:249–61. doi: 10.1016/j.omto.2022.04.009 (PMC9108396; doi:10.1016/j.omto.2022.04.009)
Supplement: Document S2. Article plus supplemental information [file mmc2.pdf]

Molecular Therapy
Oncolytics
Original Article

Modulation of *p53* expression in cancer-associated fibroblasts prevents peritoneal metastasis of gastric cancer

Toshihiro Ogawa,[1] Satoru Kikuchi,[1] Motoyasu Tabuchi,[1] Ema Mitsui,[1] Yuta Une,[1] Hiroshi Tazawa,[1,2] Shinji Kuroda,[1] Kazuhiro Noma,[1] Toshiaki Ohara,[1,3] Shunsuke Kagawa,[1] Yasuo Urata,[4] and Toshiyoshi Fujiwara[1]

[1]Department of Gastroenterological Surgery, Okayama University Graduate School of Medicine, Dentistry and Pharmaceutical Sciences, 2-5-1 Shikata-cho, Kita-ku, Okayama 700-8558, Japan; [2]Center for Innovative Clinical Medicine, Okayama University Hospital, Okayama 700-8558, Japan; [3]Department of Pathology and Experimental Medicine, Okayama University Graduate School of Medicine, Dentistry and Pharmaceutical Sciences, Okayama 700-8558, Japan; [4]Oncolys BioPharma, Tokyo 106-0032, Japan

Cancer-associated fibroblasts (CAFs) in the tumor microenvironment are associated with the establishment and progression of peritoneal metastasis. This study investigated the efficacy of replicative oncolytic adenovirus-mediated *p53* gene therapy (OBP-702) against CAFs and peritoneal metastasis of gastric cancer (GC). Higher CAF expression in the primary tumor was associated with poor prognosis of GC, and higher CAF expression was also observed with peritoneal metastasis in immunohistochemical analysis of clinical samples. And, we found transcriptional alteration of *p53* in CAFs relative to normal gastric fibroblasts (NGFs). CAFs increased the secretion of cancer-promoting cytokines, including interleukin-6, and gained resistance to chemotherapy relative to NGFs. OBP-702 showed cytotoxicity to both GC cells and CAFs but not to NGFs. Overexpression of wild-type *p53* by OBP-702 infection caused apoptosis and autophagy of CAFs and decreased the secretion of cancer-promoting cytokines by CAFs. Combination therapy using intraperitoneal administration of OBP-702 and paclitaxel synergistically inhibited the tumor growth of peritoneal metastases and decreased CAFs in peritoneal metastases. OBP-702, a replicative oncolytic adenovirus-mediated *p53* gene therapy, offers a promising biological therapeutic strategy for peritoneal metastasis, modulating CAFs in addition to achieving tumor lysis.

INTRODUCTION

The most frequent form of distant metastasis and recurrence in advanced gastric cancer (GC) is peritoneal metastasis, which is considered an independent predictor of poor prognosis and lacks curative treatment options.[1] Although peritoneal metastasis is caused by dissemination of cancer cells from the primary site into the peritoneal cavity and implantation onto mesothelial cells, the tumor microenvironment, including extracellular matrix, cancer-associated fibroblasts (CAFs), and immune cells and their interactions, enhance cancer progression, metastasis, and the form of peritoneal metastasis.[2–4] CAFs are one of the important components of the tumor mesenchyme and are known to enhance cancer progression and metastasis.[2,5] CAFs differ from normal fibroblasts (NFs) in various structural and functional aspects. The expression and function of *p53* are downmodulated in CAFs, and transcriptional alteration of *p53* converts NFs to CAFs, which become cancer-supporting rather than cancer-inhibiting.[6–8] Mutation of the *p53* gene is considered to occur in over 50% of all human cancers, because *p53* plays tumor-suppressive roles and maintains genome integrity and cellular homeostasis by regulating cell-cycle arrest, senescence, apoptosis, and autophagy.[9,10] Gene therapy to introduce the tumor suppressor *p53* gene is a promising antitumor strategy that could lead to the efficient induction of tumor cell death.[11] Clinical trials using a *p53*-expressing replication-deficient adenovirus vector (Ad-*p53*) have been performed in patients with various types of cancers, and its feasibility has been confirmed.[12–14] Moreover, *p53*-expressing conditionally replicating adenovirus vectors represent promising agents in the cancer treatment.

We have previously developed a telomerase-specific replication-competent oncolytic adenovirus, OBP-301 (suratadenoturev), which drives the *E1A* and *E1B* genes for viral replication under control of the human reverse transcriptase promoter, and have confirmed its antitumor effects in various human tumor cells.[15–17] We have recently shown that intraperitoneal (i.p.) administration of OBP-301 synergistically suppressed the peritoneal metastasis of GC in combination with paclitaxel (PTX).[18] In phase I clinical studies, OBP-301 was well tolerated by patients with various cancers.[19,20] We have further developed OBP-702 as a modification of OBP-301 that expresses the wild-type *p53* gene, so OBP-702 can suppress the viability of various types of tumor cells more efficiently compared with OBP-301 via exogenous *p53* overexpression in tumor cells.[21–23] The i.p. administration of

Received 20 November 2021; accepted 21 April 2022;
https://doi.org/10.1016/j.omto.2022.04.009.

Correspondence: Satoru Kikuchi, Department of Gastroenterological Surgery, Okayama University Graduate School of Medicine, Dentistry and Pharmaceutical Sciences, 2-5-1 Shikata-cho, Kita-ku, Okayama 700-8558, Japan.
E-mail: satorukc@okayama-u.ac.jp

This is an open access article under the CC BY license (http://creativecommons.org/licenses/by/4.0/).

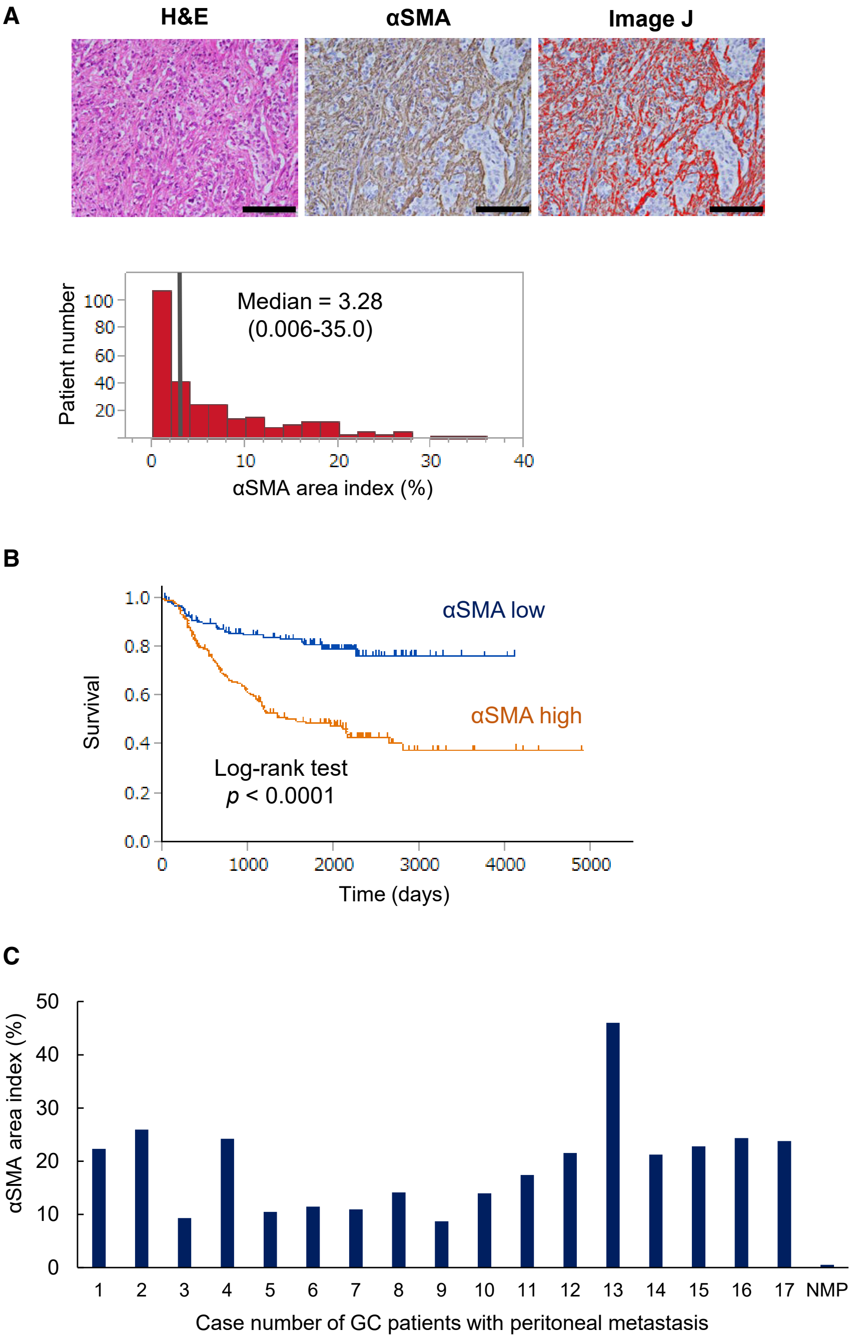

Figure 1. Analysis of α-SMA expression in clinical samples of primary gastric cancer (GC) and peritoneal metastasis by immunohistochemistry

(A) Representative microscopic images with H&E and anti-α-SMA staining, with the emphasis image from ImageJ demonstrating the evaluation of area index. Mean α-SMA-positive rate was calculated as the α-SMA area index. The α-SMA area index for each case was plotted as a histogram (black bar, median value). Scale bars, 200 μm. (B) OS curve according to α-SMA expression (high or low) in the primary tumor. The high-α-SMA expression group showed significantly worse prognosis (log rank test). (C) Enumeration of the α-SMA area index of resected peritoneal metastasis of 17 GC patients and non-metastatic peritoneal (NMP) tissue. The value of NMP indicates the mean α-SMA expression of 5 NMP tissues.

antitumor agents is advantageous for peritoneal metastasis compared with systemic therapy, because these agents can reach the peritoneal cavity directly in high concentrations.[24] In several phase III trials, i.p. chemotherapy showed superior survival benefits compared with systemic chemotherapy in patients with peritoneal metastasis of ovarian cancer.[25–27] Recently, i.p. chemotherapy showed the possibility of improved survival compared with systemic chemotherapy in a phase III trial for patients with peritoneal metastasis of GC.[28]

In this study, we found that transcriptional alteration of *p53* in CAFs contributed to the tumor-supportive features of CAFs in peritoneal metastasis of GC. Furthermore, i.p. administration of OBP-702 showed significant antitumor effects against peritoneal metastasis by modulating CAFs in addition to tumor lysis via exogenous *p53* overexpression.

RESULTS

Higher CAF expression is a poor prognostic factor and is essential for peritoneal metastasis

To investigate the clinical impact of CAFs in GC, we evaluated associations with prognosis in 280 consecutively enrolled cases of GC. CAFs in primary tumor were identified as stromal cells expressing α-smooth muscle actin (α-SMA) using immunohistochemistry. The mean value was calculated as an α-SMA area index. Area index was calculated in three sites for each tissue, and the average was calculated (Figure 1A). Similarly, eleven non-cancerous gastric tissues were investigated to determine the basal level of α-SMA expression in the stomach. The mean α-SMA area index of non-cancerous gastric tissues was 1.52 ± 0.71%. We evaluated the relationship between CAFs and the prognosis of patients with GC. Patients with high α-SMA expression showed significantly shorter overall survival than those with low expression (Figure 1B). These results suggest that CAFs in the tumor microenvironment are related to worse prognosis of GC.

To explore the tumor microenvironment in peritoneal metastasis of GC, we conducted immunohistochemical analysis of surgically resected peritoneal disseminated nodules from 17 GC patients. In all samples, α-SMA and FAP (as known CAF markers) and

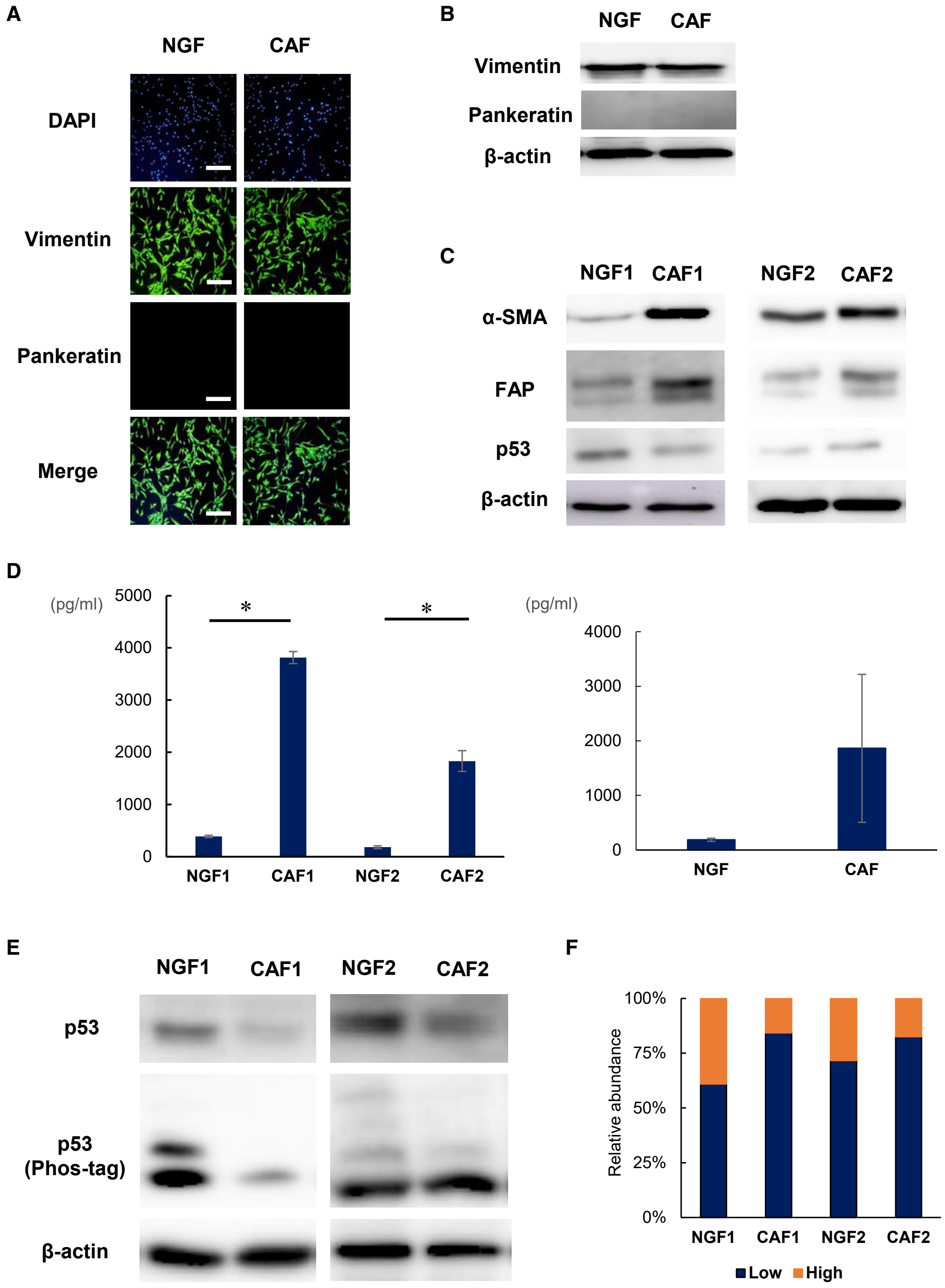

Figure 2. Hypo-phosphorylation of *p53* in CAFs regulates CAF-specific properties

(A) Immunofluorescence analysis of NGFs and CAFs established from clinical specimens. Blue color indicates the nucleus; green color indicates vimentin; and red color indicates pankeratin. Scale bar, 200 μm. (B) Western blotting analysis of NGFs and CAFs established from clinical specimens, with β-actin as a loading control. (C) Expression of α-SMA and FAP, as CAF markers, and *p53* proteins in NGFs and CAFs. β-actin was used as a loading control. (D) IL-6 secretion in NGFs and CAFs. Data are shown as

(legend continued on next page)

interleukin-6 (IL-6) were highly expressed in the fibroblasts surrounding cancer cells (Figure S1). CAF expression in peritoneal metastasis was calculated according to the analysis of the primary tumor. Similarly, non-metastatic peritoneal tissue was investigated as the basal level of α-SMA expression. The mean α-SMA area index of 5 non-metastatic peritoneal tissues was 0.51 ± 0.29%, which was considerably low, whereas higher CAF expression was confirmed in all 17 patients with peritoneal metastasis (Figure 1C). These results suggest that CAFs are related to tumor progression, metastasis, and the form of peritoneal metastasis.

Transcriptional alteration of *p53* regulates CAF-specific properties

To investigate CAF-specific properties, we isolated paired NGFs and CAFs derived from the excised stomach wall of the same GC patients (NGF1 and CAF1: poorly differentiated adenocarcinoma and NGF2 and CAF2: signet ring cell carcinoma) and cultured *in vitro*. These established fibroblasts were confirmed by immunofluorescence and western blotting analysis to express not an epithelial marker, but the mesenchymal marker vimentin, which is highly expressed in CAFs (Figures 2A and 2B). CAF markers, such as α-SMA and FAP, were highly expressed in the established CAFs (Figure 2C). Secretion of IL-6 was significantly increased in CAF1 and CAF2 compared with NGFs, respectively. Moreover, we isolated two more paired NGFs and CAFs (CAF3: poorly differentiated adenocarcinoma and CAF4: poorly differentiated adenocarcinoma) and compared IL-6 secretion between NGFs and CAFs. After all, IL-6 secretion was significantly increased in CAFs (Figure 2D). NGF2 activated by CM obtained from MKN7 or TGF-β also showed CAF-like properties (Figure S2). To explore what causes these CAF-specific properties, we investigated the phosphorylation of *p53* in CAFs and NGFs using western blot analysis in Phos-tag gel. The cellular functions of *p53* are thought to be mainly controlled by posttranslational modifications, such as phosphorylation. Interestingly, the phosphorylation of *p53* was decreased in CAFs compared with NGFs (Figures 2E and 2F). The decreased *p53* phosphorylation was associated with altered protein conformation. This result suggests that transcriptional alteration of *p53* in CAFs might contribute to their specific properties.

Synergistic antitumor effect of OBP-702 and PTX on human gastric cancer cells

OBP-702 is a modified oncolytic adenovirus, in which wild-type *p53* gene was inserted into the E3 region in OBP-301. To evaluate the antitumor effects of OBP-702 and PTX, MKN45 or NUGC4 cells that are resistant to OBP-301 (Figure S3) were treated with OBP-702 or PTX. Cell death was induced in both MKN45 and NUGC4 cells in a dose-dependent manner following treatment with OBP-702 or PTX (Figure 3A). OBP-702 infection induced apoptosis (as confirmed by the accumulation of cleaved PARP) and stimulated autophagy (as confirmed by p62 downregulation) in human GC cells (Figure 3B). The XTT cell viability assay demonstrated that combination therapy using OBP-702 and PTX induced cancer cell death in a dose-dependent manner. Calculation of the combination index indicated a synergistic antitumor effect of combination therapy in both types of human GC cells (Figure 3C). We reported the chemosensitizing effect of OBP-301, which has the same basic structure as OBP-702, except for *p53* in several types of human malignant tumor cells.[29,30] We recently showed that PTX enhanced the replication efficiency of OBP-301 in cancer cells, and combination therapy increased the mitotic catastrophe of cancer cells, resulting in synergistic antitumor effects.[18] These results suggest that the combination of OBP-702 and PTX has a stronger synergistic antitumor effect on human GC cells.

OBP-702 has a CAF-selective cytotoxic effect via wild-type *p53*

Although PTX shows cytotoxicity to NGF in a dose-dependent manner, CAFs were confirmed to acquire chemoresistance in an XTT cell viability assay (Figure 4A). The OBP-301 and OBP-702 oncolytic adenoviruses can replicate selectively only within human cancer cells via human telomerase reverse transcriptase (hTERT) promoter. OBP-301 and OBP-702 did not affect the viability of NGFs, which are normal cells. Interestingly, although OBP-301 did not show cytotoxicity to CAFs, OBP-702 was cytotoxic to CAFs in a dose-dependent manner in an XTT cell viability assay (Figures 4A and S4). NGF2 activated by TGF-β showed CAF-like properties, which were OBP-702-sensitive and PTX-resistant (Figure S5). Seventy-two hours after OBP-702 infection at 100 multiplicities of infection (MOIs), the number of living CAFs was also decreased compared with NGFs in the morphological observation (Figure 4B), although hTERT expression was similar between NGFs and CAFs in a real-time PCR assay (Figure S6). OBP-702 infection to CAFs were confirmed by the accumulation of viral E1A protein in a dose-dependent manner, and OBP-702 infection induced the phosphorylation of *p53*, resulting in the induction of apoptosis and autophagy in CAFs (Figure 4C). With the cell death of CAFs caused by OBP-702, secretion of IL-6 from CAFs was decreased, although this was not observed in OBP-301 infection (Figure 4D). Similarly, secretions of other cancer-promoting cytokines, such as C-X-C motif chemokine ligand (CXCL)5, CXCL1, CCL8, and CCL7 from CAFs, were decreased by OBP-702 infection, although these cytokines were increased in CAFs compared with NGFs (Figure 4E). These results suggest that OBP-702 has selective cytotoxic effects on CAFs by the induction of wild-type *p53*.

Intraperitoneal virotherapy combined with PTX for peritoneal metastasis

Next, we examined whether i.p. administration of OBP-702 combined with PTX could eradicate peritoneal metastasis using a MKN45-Luc xenograft mouse model. OBP-702 or PBS was injected i.p. 10 days after tumor inoculation. PTX was injected i.p. 2 days after OBP-702 injection

mean ± SD. Statistical significance was defined as $p < 0.05$ (*). (Left) The comparison of IL-6 secretion between two pairs of NGFs and CAFs. (Right) The comparison of IL-6 secretion between the average of four pairs of NGFs and CAFs. (E) Extracts from NGFs and CAFs were separated by standard SDS-PAGE (top) and 30 μM Phos-tag SDS-PAGE (bottom), followed by western blot analysis with *p53* antibodies. β-actin was used as a loading control. (F) The relative abundance of each band in the Phos-tag gel was compared between NGFs and CAFs.

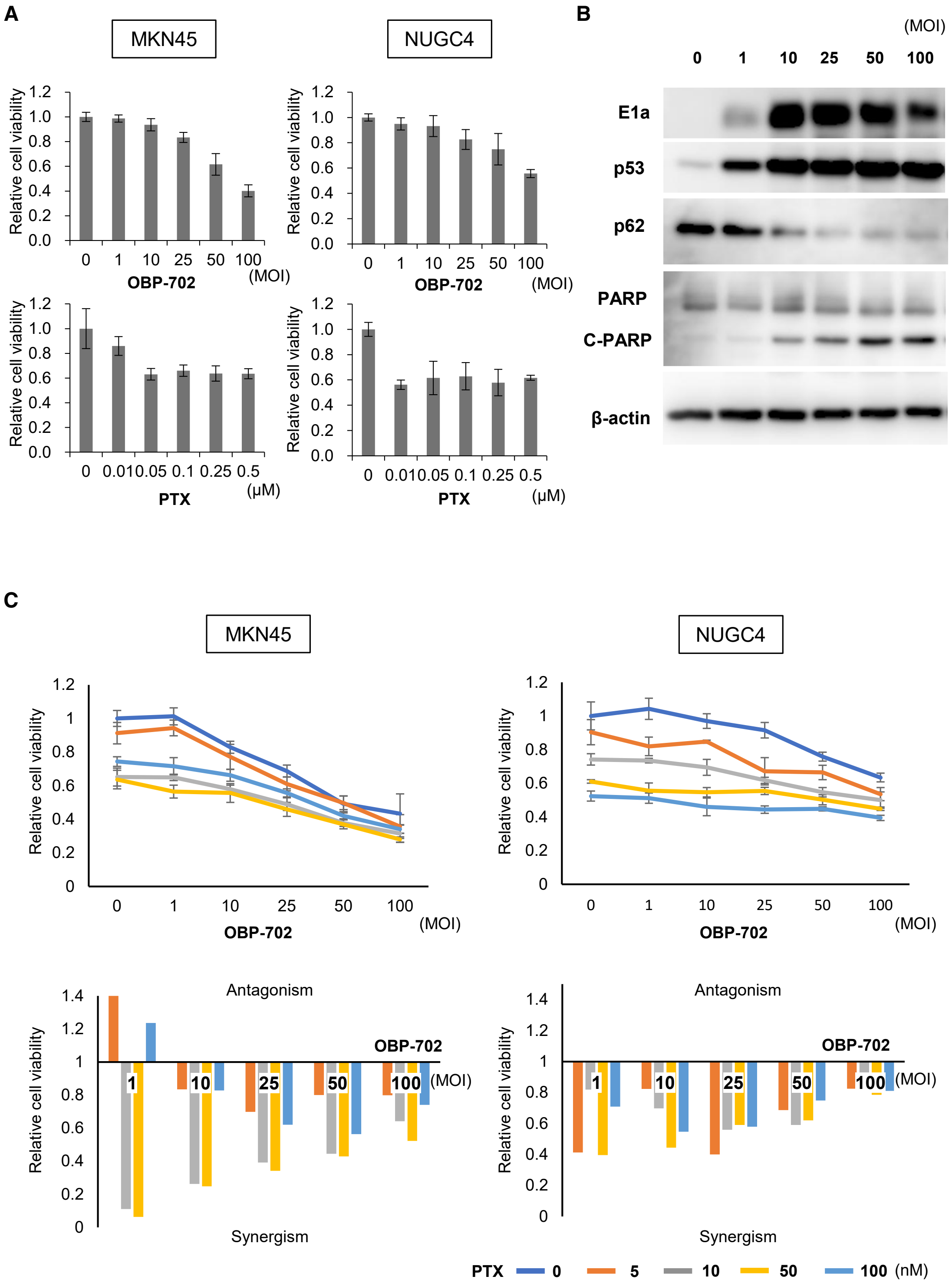

Figure 3. Combination of *p53*-expressing OBP-702 and PTX shows synergistic antitumor effects on human gastric cancer cells

(A) MKN45 and NUGC4 cells were infected with OBP-702 at the indicated MOIs for 3 days. Cells were treated with PTX at the indicated doses for 24 h. Cell viability was quantified using the XTT assay. The cell viability of a mock-treated group was considered 1.0, and relative cell viabilities were then calculated. Data are expressed as mean ± SD (n = 5). (B) Expression of PARP, C-PARP, p62, *p53*, and adenoviral E1A proteins in MKN45 cells infected with OBP-702 at the indicated MOIs. β-actin was used as a loading control. (C) The combination index was calculated with CalcuSyn software. Synergism and antagonism were defined as interaction indices of <1 and >1, respectively.

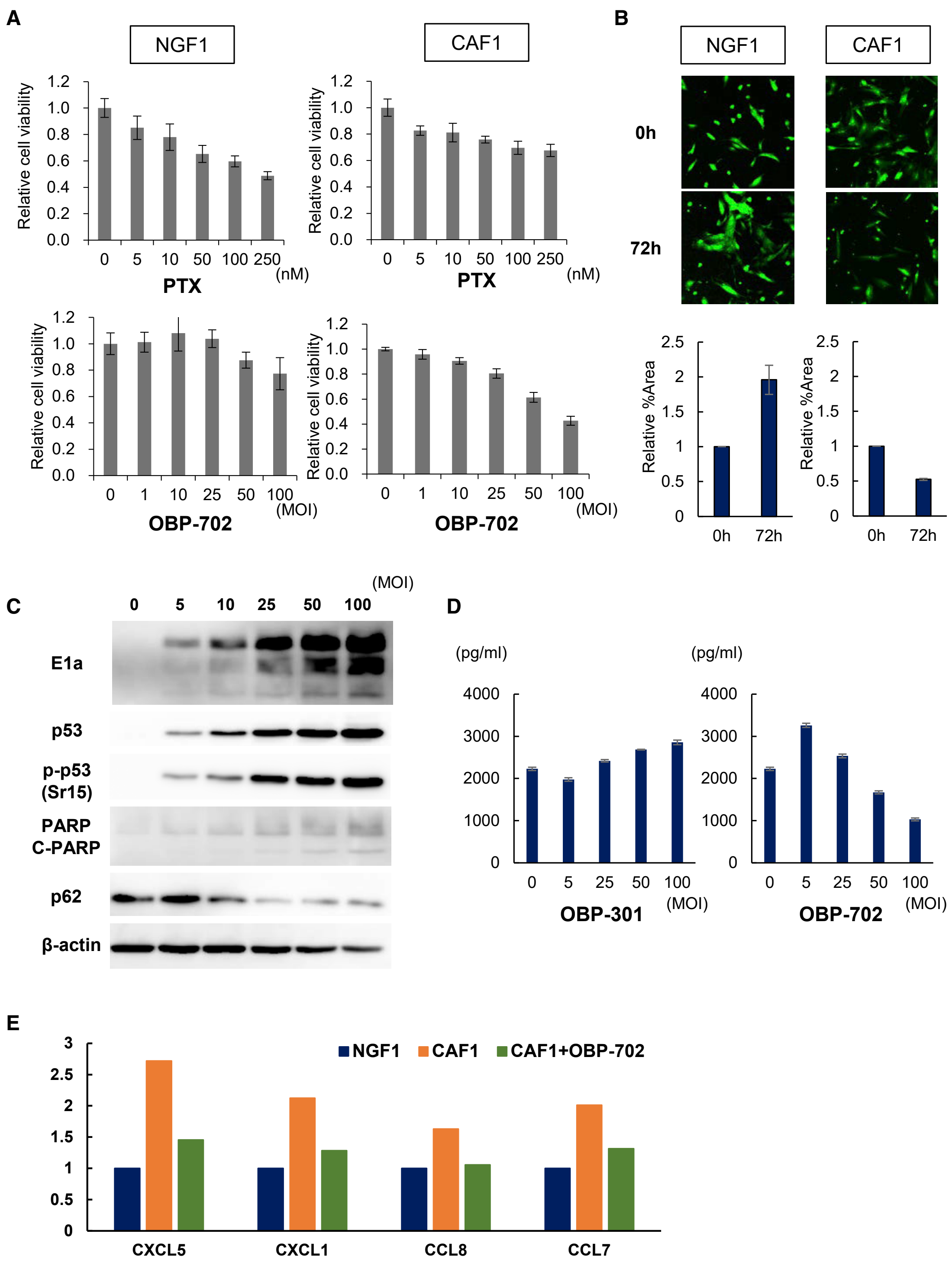

Figure 4. OBP-702 suppresses the function and viability of CAFs by inducing *p53*

(A) NGFs and CAFs were infected with OBP-702 at the indicated MOIs for 3 days. Cells were treated with PTX at the indicated doses for 24 h. Cell viability was quantified using the XTT assay. The cell viability of a mock-treated group was considered 1.0, and relative cell viabilities were calculated. Data are expressed as mean ± SD (n = 5). (B) Time-lapse images of NGF and CAF infected with OBP-702 for 72 h. Cells were stained by CellTracker™. Relative ratios of the green area at 72 h after viral infection as compared

(legend continued on next page)

for only one cycle (Figure 5A). The combination of OBP-702 and PTX significantly suppressed i.p. tumor growth compared with PBS or monotherapy with either OBP-702 or PTX (Figures 5B and 5C). This result suggests that i.p. virotherapy using OBP-702 combined with PTX has a stronger antitumor effect on peritoneal metastases from GC.

Combination therapy using OBP-702 and PTX eradicates peritoneal metastasis by suppressing both cancer cells and CAFs

To evaluate whether CAFs contribute to tumor growth in the peritoneal cavity, we inoculated cancer cells (MKN45-Luc) or co-inoculated cancer cells and CAF (MKN45-Luc + CAF2) into the peritoneal cavity of BALB/c nu/nu mice and compared the tumor growth of MKN45-Luc between groups. CAFs enhanced tumor growth in the peritoneal cavity (Figure 6A). Next, we evaluated the effects of combination therapy with OBP-702 and PTX for a peritoneal metastasis model co-inoculated with CAF. OBP-702 was administered i.p. 3 times each week, and PTX was also administered i.p. 2 days after OBP-702 administration (Figure 6B). Combination therapy significantly suppressed total tumor weight in the peritoneal cavity (Figure 6C), whereas OBP-301 treatment did not suppress tumor weight significantly (Figure S7). Furthermore, immunohistochemical analyses demonstrated that combination therapy significantly decreased the α-SMA-expressing fibroblasts in peritoneal tumors compared with monotherapy (Figures 6D and 6E). These results suggest that i.p. combination therapy using OBP-702 and PTX has multipotent therapeutic effects for the peritoneal metastasis of GC by the suppression of both cancer cells and CAFs.

DISCUSSION

In the tumor microenvironment (TME) of peritoneal metastasis, CAFs are stromal fibroblasts on adjacent cancer cells that play important roles in implanting cancer cells and tumor growth [2-4]. Suppressing non-cancerous cells, such as CAFs, in addition to cancer cells is important to eradicate intractable peritoneal metastasis. This study demonstrated that CAFs acquired tumor-supportive properties by the transcriptional alteration of *p53* in CAFs. Furthermore, we showed the therapeutic potential of i.p. administration of OBP-702 in combination with PTX for peritoneal metastasis of GC by modulating CAFs in addition to tumor lysis.

Although peritoneal metastasis is the typical and most frequent pattern of metastasis and recurrence in advanced GC, the mechanisms underlying peritoneal metastasis remain unclear. The seed and soil theory has become established as the fundamental theory of peritoneal metastasis.[31,32] In this theory, cancer cells are compared with "seeds" and the TME in the peritoneal site to "soil." The key factors interacting in the TME are CAFs and immunocytes, such as tumor-associated macrophages (TAMs).[32,33] We have previously shown that i.p. TAMs play important roles in the formation and progression of peritoneal metastasis of GC via secreted IL-6. CAFs have also been reported as the major sources of IL-6 in TME, similar to TAMs.[34,35] In immunohistochemical analyses of surgically resected peritoneal disseminated nodules of GC patients, we demonstrated that both CAFs and IL-6 were highly expressed in all cases, suggesting that CAFs are indispensable to establish peritoneal metastasis (Figure 1). CAFs stimulated by some cytokines secreted by cancer cells also release several tumor-progressive cytokines, such as IL-6.[4,36] IL-6 in the TME has been reported to play an important role in tumor progression and chemoresistance via activation of the JAK/STAT3 pathway.[37,38] In our study, CAFs established from clinical samples showed higher α-SMA expression and IL-6 secretion compared with NGFs (Figure 2). Originally, NFs tend to suppress tumor progression and maintain tissue homeostasis. In addition, *p53* plays a tumor-suppressive role in fibroblasts by inhibiting the production and secretion of tumor-progressive factors.[39,40] However, continued interaction with cancer cells converts adjacent fibroblasts into CAFs.[2] The distinctive cancer-supportive properties of CAFs have been attributed to epigenetic modifications.[6–8] Although CAFs are commonly believed to not have *p53* mutations, *p53* is functionally compromised in CAFs.[8,41] We have shown that CAFs revealed selective reductions in phosphorylated forms of *p53* (Figure 2). This alteration of *p53* phosphorylation in CAFs might contribute to transcriptional reprograming of the normal tumor-suppressive nature of *p53* into its tumor-supporting nature in CAFs. Because *p53* is one of the most frequent targets for mutational inactivation in the various cancers,[10] targeting *p53* by gene therapies would be an effective treatment strategy that could influence not only cancer cells but also CAFs in TME. An oncolytic adenovirus, OBP-702, expresses the wild-type *p53* gene and so induces apoptosis and autophagy in various types of cancer cells.[21,22] Because *p53* is a strong inducer of autophagy as well as apoptosis, OBP-702 also induced apoptosis and autophagy in CAFs via the induction of wild-type *p53* (Figure 4C). Although replication of the oncolytic adenovirus we used is driven by the hTERT promoter, expression of hTERT in CAF was similar to that in NGFs (Figure S4). Moreover, OBP-301 did not affect the viability of either NGFs or CAFs *in vitro* and could not suppress peritoneal metastasis *in vivo*. These results suggest that induction of wild-type *p53* by OBP-702 leads to apoptosis and autophagy in CAFs.

In preclinical studies, we found that oncolytic adenovirus enhanced the antitumor effects of chemotherapeutic agents in several human cancer cells.[31,42–44] In particular, PTX is suitable for combination use with oncolytic adenovirus, because DNA synthesis in host cells is not inhibited. Recently, we have shown that the combination of PTX and oncolytic adenovirus had synergistic antitumor effects in

with 0 h were calculated. (C) Expressions of *p53*, p-*p53*, adenoviral E1A, PARP, and p62 proteins in CAFs infected with OBP-702 at the indicated MOIs. β-actin was used as a loading control. (D) Amounts of IL-6 secreted from CAF1 4 days after OBP-301 or OBP-702 infection were quantified by ELISA. Data are expressed as mean ± SD (n = 3). (E) Changes in tumor-progressive cytokines secreted from CAFs 3 days after infection with 10 MOIs of OBP-702. Cytokines secreted from NGF as the control were analyzed. Intensities of cytokines were measured by ImageJ. The amount of cytokines secreted from NGF was considered 1.0; then, relative amounts of cytokines secreted from CAF were calculated.

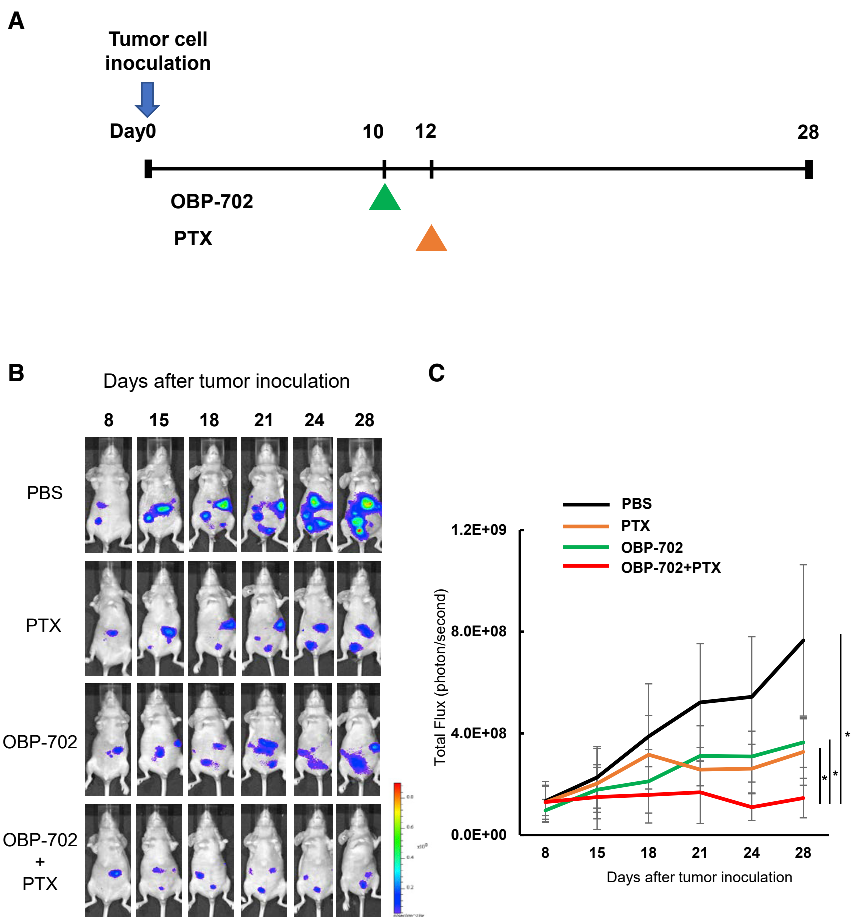

Figure 5. Peritoneal metastasis of GC was suppressed by i.p. administration of OBP-702 in combination with PTX in the orthotopic mouse model

(A) Study protocol. The i.p. tumors of MKN-45-Luc were treated with i.p. OBP-702 (1×10^5 PFUs) and/or i.p. PTX (10 mg/kg body weight). (B) Representative photographs of mice with peritoneal metastasis treated with PBS, OBP-702, PTX, or OBP-702 and PTX. (C) Luminescence in tumor tissues was analyzed using the IVIS system at 15, 18, 21, 24, and 28 days after tumor inoculation. Data are expressed as mean value ± SD (n = 5). Statistical significance was defined at the level of $p < 0.05$ (*).

GC cells, because PTX enhanced the replication efficacy of oncolytic adenovirus, and combination therapy increased the induction of mitotic catastrophe, a distinctive process of cell death caused by abnormal mitosis in cancer cells.[18] PTX is also a suitable chemotherapeutic agent administered i.p. for peritoneal metastasis, given PTX can be retained in the peritoneal cavity for a long time, due to its molecular characteristics. A phase III trial using i.p. administration of PTX has suggested the possibility of improved survival in GC patients with peritoneal metastasis.[28] In this study, combination therapy using i.p. administration of PTX and OBP-702 significantly suppressed the tumor growth of peritoneal metastasis.

A phase I clinical trial of intratumoral injection of OBP-301 was conducted in patients with a variety of advanced solid tumors in the United States, and the safety and feasibility of OBP-301 have already been confirmed.[19] We recently reported that intratumoral OBP-301 injection with radiotherapy was feasible and showed clinical benefits in patients with esophageal cancer unfit for standard treatments.[20] Some phase I trials of i.p. administration of oncolytic virus for patients with ovarian cancer have been conducted and the safety profiles also confirmed.[45,46] In a preclinical study, we confirmed that oncolytic adenovirus administered i.p. is distributed to i.p. metastatic nodules in a xenograft model.[18] In this study, i.p. administration of OBP-702 combined with PTX suppressed the proliferation of CAFs in addition to tumor growth in the peritoneal cavity. The combination of OBP-702 and PTX might be an optimal treatment strategy for cases showing peritoneal metastasis of GC, because this combination therapy could not only affect cancer cells but also CAFs adjacent to cancer cells.

In conclusion, we demonstrated that transcriptional alteration of *p53* in CAFs might regulate tumor-supportive properties. OBP-702 suppressed not only cancer cells but also CAFs by inducing wild-type *p53*. Further, i.p. administration of OBP-702 and PTX showed significant antitumor effects against peritoneal metastasis in an orthotopic xenograft model by suppressing both cancer cells and CAFs. Our oncolytic adenovirus-mediated *p53* gene therapy in combination with PTX offers promise as a biological therapeutic strategy for peritoneal metastasis from GC.

MATERIALS AND METHODS

Patients and immunohistochemistry in clinical samples

A total of 280 patients with GC who received gastrectomy at Okayama University Hospital between 2002 and 2009 were retrospectively investigated and reviewed. Seventeen patients with peritoneal metastasis of GC who underwent resection of peritoneal nodules between 2014 and 2018 were investigated. First, the presence of tumor was confirmed using H&E staining. Sectioned tissues were incubated with rabbit anti-α-SMA monoclonal antibody (mAb) (Sigma-Aldrich, St. Louis, MO, USA), rabbit anti-fibroblast activation protein (anti-FAP) polyclonal antibody (pAb), or mouse anti-IL-6 mAb (Abcam, Cambridge, MA, USA) for immunohistochemistry. CAFs were defined as spindle-shaped cells expressing α-SMA, and α-SMA scoring was evaluated using an area index, calculated in low-magnification fields by ImageJ software (http://rsb.info.nih.gov/ij/). Four fields, including stromal cells per sample, were carefully selected to evaluate CAFs. The mean value obtained from each sectioned tissue was defined as the α-SMA area index. All evaluations were performed

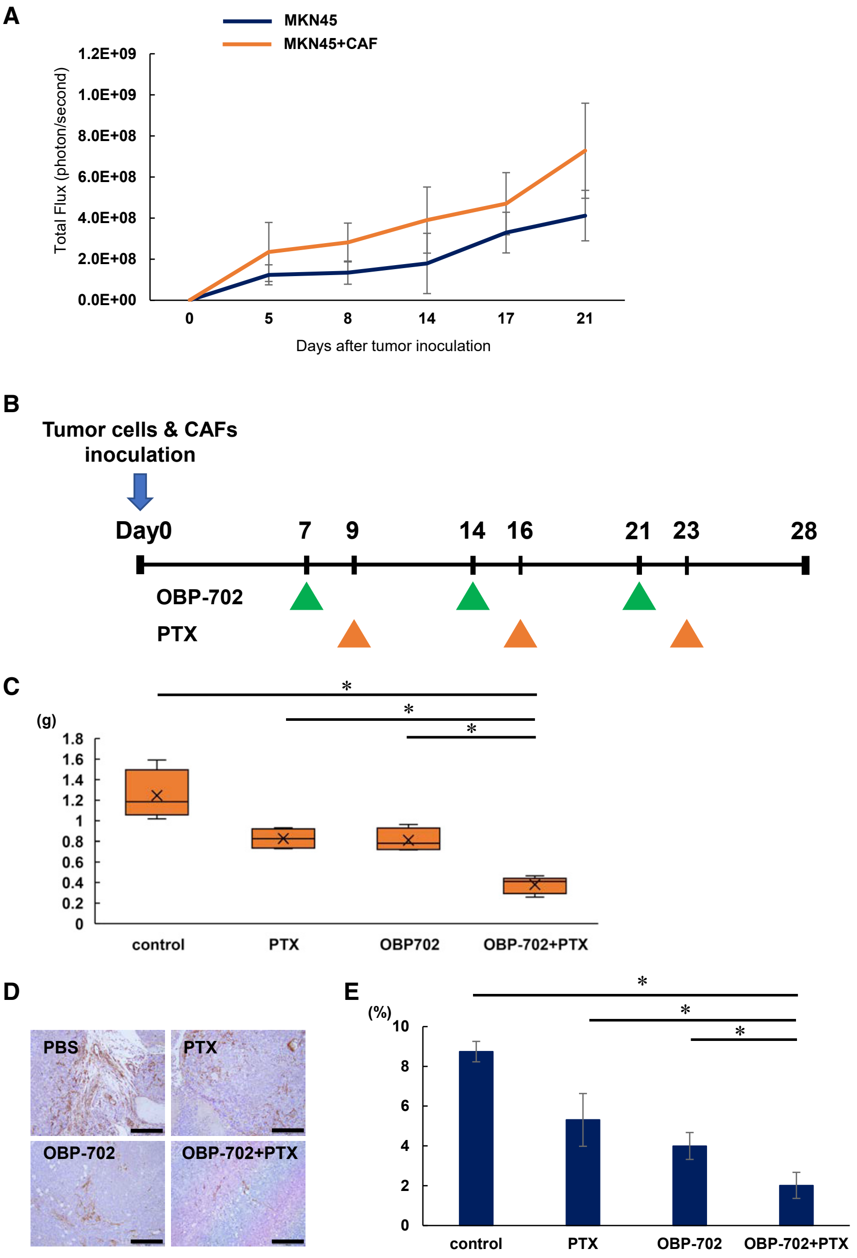

Figure 6. Tumor growth was suppressed by i.p. administration of OBP-702 in combination with PTX via suppression of both cancer cells and CAFs in metastatic sites

(A) MKN45-Luc (5 $\times 10^6$ cells) or co-inoculated with CAFs (2.5 $\times 10^6$ cells) were inoculated into the abdominal cavity of nude mice. Luminescence in tumor tissue was analyzed using the IVIS system at 5, 8, 14, 17, and 21 days after tumor inoculation. Data are expressed as mean value ± SD (n = 5). (B) Study protocol. i.p. tumors of MKN-45-Luc and CAFs were treated with OBP-702 (1 $\times$ 10^7 PFUs) i.p. and/or PTX (10 mg/kg body weight) i.p. three times each week. (C) All tumor nodules in the abdominal cavity were resected and total volume weight was measured on day 28. Data are expressed as mean ± SD (n = 4). Statistical significance was defined as $p < 0.05$ (*). (D) Histological analysis for α-SMA protein expression of peritoneal tumors. Tumor tissues were obtained on day 28. Scale bars, 200 μm. (E) The α-SMA-expressing areas in the peritoneal tumors were evaluated using an area index, calculated by ImageJ software. Data are expressed as mean ± SD (n = 4). Statistical significance was defined as $p < 0.05$ (*).

by an independent pathologist blinded to clinical information. Immunoreactive signals were visualized with a 3,3'-diaminobenzidine tetrahydrochloride solution, and nuclei were counterstained with hematoxylin. Sections were observed under light microscopy (BX50; Olympus, Tokyo, Japan).

Cell lines

This study used the four human GC cell lines, MKN7, NUGC4, MKN45, and MKN45-Luc, which were transfected with the firefly luciferase plasmid vector. These cells were purchased from the Japanese Collection of Research Bioresources Cell Bank and maintained in RPMI-1640 medium supplemented with 10% heat-inactivated fetal bovine serum (FBS) (Sigma-Aldrich). Primary human esophageal fibroblasts, designated FEF3, were isolated from human fetal esophagus as described previously.[47] CAFs were established from the surgically excised gastric wall of the tumor and normal gastric fibroblasts (NGFs) were from non-tumoral gastric wall tissue. CAFs and NGFs were maintained DMEM with 10% FBS and 0.5 mM sodium pyruvate. Written informed consent was obtained from patients prior to the study. All media were supplemented with 100 U/mL penicillin and 100 mg/mL streptomycin. Cells were routinely maintained at 37°C in a humidified atmosphere with 5% CO_2.

Recombinant adenovirus and reagents

The recombinant, telomerase-specific, replication-competent adenovirus vector OBP-301 (suratadenoturev) has been described and characterized previously.[15–17] OBP-702 is an adenovirus variant that inserts a human wild-type *p53* gene expression cassette under control of the Egr-1 promoter into the E3 region in OBP-301. *E1A*-deleted adenovirus vector lacking a cDNA insert (dl312) and wild-type adenovirus type 5 were also used as control vectors. Viruses were purified by ultracentrifugation using cesium chloride step gradients. Viral titers were determined by a plaque-forming assay using 293 cells, and the virus was stored at −80°C. PTX was purchased from Nippon Kayaku (Tokyo, Japan) and dissolved in PBS. Recombinant human transforming growth factor β1 (TGF-β1) was obtained from Sigma-Aldrich.

Cell viability assay

All cells were seeded on 96-well plates at a density of 1×10^3 cells/well and cultured for 24 or 72 h before viral infection or administration of PTX. Cell viability was examined 96 h after cell seeding using a Cell Proliferation Kit II (Roche Molecular Biochemicals, Indianapolis, IN, USA), which is based on a sodium 3'-[1-(phenylaminocarbonyl)-3,4-tetrazolium]-bis(4-methoxy-6-nitro) benzene sulfonic acid hydrate (XTT) assay, according to the protocol from the manufacturer. The combination index was calculated with CalcuSyn software (BioSoft, Cambridge, UK). Computation of the combination index was based on the methods of Chou and Talalay.[48]

Immunofluorescence

Cells were seeded at a density of 2×10^4 cells/mL for 24 h. Following three washes with PBS, cells were fixed in 100% methanol for 30 min at room temperature (RT). After blocking endogenous peroxidases, cells were incubated with Alexa Fluor® 647 antivimentin antibody (Abcam) and mouse anti-pankeratin mAb (Cell Signaling Technology, Danvers, MA, USA) overnight at 4°C. Following three washes with PBS, cells were incubated with Alexa Fluor® 647 (Invitrogen, Carlsbad, CA, USA) as secondary antibody for 60 min at 4°C. After washing, nuclei were stained with DAPI (Invitrogen) for 3 min. Cells were viewed under light microscopy (IX83; Olympus).

Flow-cytometric analysis

Cells were washed with PBS containing 2% FBS and centrifuged at 300*g*, 4°C, for 5 min and then incubated with primary anti-CAR antibody (Sigma-Aldrich) for 60 min at 4°C. After washing the same way, cells were incubated with secondary fluorescein-conjugated anti-mouse antibody (Invitrogen) for 30 min at 4°C in darkness. As controls, immunoglobulin G (IgG)-isotype control antibodies (Santa Cruz Biotechnology, Dallas, TX, USA) were used. Cells were analyzed by flow cytometry (FACSArray; BD Biosciences, San Jose, CA, USA), and data were reanalyzed using FlowJo software (BD Biosciences, Franklin Lakes, NJ, USA).

Quantitative real-time PCR analysis

Total RNA (48 h after incubation) was isolated from cells using the RNeasy Mini Kits (QIAGEN, Hilden, Germany), according to the instructions from the manufacturer. The cDNA was synthesized from 1.0 mg of total RNA using Advantage RT-for-PCR Kit (Clontech Laboratories, Mountain View, CA, USA). Quantitative real-time PCR was performed for gene expression analysis using the StepOnePlus Real-Time PCR System (Applied Biosystems, Waltham, MA, USA) with Taqman PCR master mix (Applied Biosystems, Foster City, CA, USA). The primers were GAPDH (Applied Biosystems, Waltham, MA, USA) and hTERT (Applied Biosystems, Waltham, MA, USA). GAPDH was used as a normalization control. The relative expression of each mRNA was determined using the ΔΔCt method.

Time-lapse imaging

NGFs and CAFs were seeded in a 27-mm glass-based dish at a density of 3×10^4 cells/well and stained with 10 μM CellTracker™ Green CMFDA Dye (Invitrogen) for 30 min at 37°C in the absence of FBS. After exchange to normal medium, cells were infected with OBP-702 at 100 MOIs. Time-lapse images were taken serially for 72 h after viral infection using a confocal laser scanning biological microscope with built-in culture incubator (FV10i; Olympus). Green area was evaluated using an area index, calculated by ImageJ software.

Western blot analysis

MKN45 cells seeded in a 100-mm dish at a density of 3×10^5 cells/dish were infected with OBP-702 at the indicated MOIs for 72 h. Whole-cell lysates were prepared in lysis buffer (50 mM Tris-HCl [pH 7.4], 150 mM NaCl, and 1% Triton X-100) containing a protease inhibitor cocktail (cOmplete Mini; Roche Applied Science, Mannheim, Germany) and phosphatase inhibitor cocktail (PhosSTOP; Roche Applied Science). Proteins were separated by SDS-PAGE and transferred to polyvinylidene difluoride membranes (Hybond P; GE Healthcare, Chicago, IL, USA). Membranes were blocked with Blocking One (Nacalai Tesque, Kyoto, Japan) at RT for 30 min and then incubated overnight at 4°C with the following antibodies: mouse anti-Ad5 E1A mAb (BD Pharmingen, Franklin Lakes, NJ, USA), mouse anti-*p53* mAb (DO-1), rabbit anti-p-*p53* pAb, rabbit anti-PARP pAb, rabbit anti-SQSTM1/p62 pAb, rabbit anti-α-SMA mAb, mouse anti-pankeratin mAb (Cell Signaling Technology), rabbit anti-FAP pAb, rabbit anti-vimentin mAb (Abcam), and mouse anti-β-actin mAb (Sigma-Aldrich). Immunoreactive bands on blots were visualized using enhanced chemiluminescence substrates (ECL Plus; GE Healthcare). For the phosphorylation of *p53* in NGFs and CAFs, cell lysates were prepared in the lysis buffer containing EDTA-free protease inhibitor cocktail and PhosSTOP. Phos-tag (Wako Pure Chemical, Osaka, Japan) gels were prepared and run, according to the protocol from the manufacturer. Mixed antibodies between DO-1 and PAb1801 (Abcam) were used as primary antibodies of *p53*.

Cytokine array

Cells were seeded at a density of 10×10^4 cells/mL and infected with OBP-702 at 100 MOIs 24 h after cell seeding. Supernatants were collected 96 h after virus infection. Cytokine and chemokine levels were measured using Cytokine Array (Abcam), according to the protocol from the manufacturer. Immunoreactive spots were visualized using enhanced chemiluminescence substrates (ECL Plus). Comparison between samples was performed using ImageJ software.

ELISA

Cells were seeded at a density of 4×10^4 cells/mL and infected with OBP-702 at 100 MOIs 24 h after cell seeding. Supernatants were collected 72 h after virus infection. IL-6 levels in cell culture supernatant were measured using a Human IL-6 Quantikine ELISA Kit (R&D Systems, Minneapolis, MN, USA), according to the protocol from the manufacturer.

Animal experiments

MKN45-Luc cells (5×10^6 cells) were inoculated into the peritoneal cavity of 7-week-old female BALB/c nu/nu mice (CLEA Japan, Tokyo, Japan), as models of peritoneal dissemination. Ten days after

cell inoculation, 500 μL of solution containing OBP-702 (1×10^5 plaque-forming units [PFUs]) or PBS was injected i.p. and PTX (10 mg/kg body weight) was also injected i.p. 2 days after OBP-702 injection. Five mice were used for each group. To monitor tumor progression, the substrate luciferin (VivoGlo Luciferin; Promega, Madison, WI, USA) was injected i.p. at a dose of 200 mg/kg body weight. Images were collected in the supine position every few minutes after luciferin injection with the Lumina *in vivo* imaging system (IVIS) imaging system (Caliper Life Sciences, Cheshire, UK), and photons emitted from the abdominal cavity were quantified using Xenogen Living Image software (Caliper Life Sciences). In the co-injection model, both MKN45-Luc (5×10^6 cells) and CAFs (2.5×10^6 cells) were inoculated i.p. into nude mice. OBP-702 (1×10^7 PFUs), or PBS was injected i.p. every week (days 7, 14, and 21), and PTX (10 mg/kg body weight) was also injected i.p. 2 days after virus injection (days 9, 16, and 23). All tumor nodules in the peritoneal cavity were resected and total weights were measured on day 28.

Anti-α-SMA mAb (Sigma-Aldrich) was used for immunohistochemical investigation of peritoneal tumor nodules. Immunoreactive signals were visualized with a 3,39-diaminobenzidine tetrahydrochloride solution, and nuclei were counterstained with hematoxylin. Sections were viewed under light microscopy (BX50; Olympus).

Statistical analysis

For the area index of α-SMA, cutoff was defined using the median value of the high or low groups. Overall survival (OS) was calculated using the Kaplan-Meier method, with the log rank test used for comparisons between subgroups. Student's t test was used to identify statistically significant differences between groups. All data are expressed as means ± SD. Values of $p < 0.05$ were considered statistically significant. Statistical analysis was performed using JMP, v.11.2 (SAS Institute, Cary, NC, USA).

Study approval

This study was conducted in accordance with the ethical standards of the Declaration of Helsinki and the ethical guidelines for medical and health research involving human subjects. Studies using clinical samples were approved and reviewed by the institutional review board of Okayama University Hospital (approval nos. 1707-022 and 1505-023). All animal experimental protocols were approved by the Ethics Review Committee for Animal Experimentation of Okayama University (approval no. OKU-2020166).

SUPPLEMENTAL INFORMATION

Supplemental information can be found online at https://doi.org/10.1016/j.omto.2022.04.009.

ACKNOWLEDGMENTS

We thank Ms. Tomoko Sueishi, Ms. Tae Yamanishi, and Ms. Yuko Hoshijima for their excellent technical support. Yasuo Urata is the president and CEO of Oncolys BioPharma, the manufacturer of OBP-301 and OBP-702. Hiroshi Tazawa and Toshiyoshi Fujiwara are consultants for Oncolys BioPharma. The other authors have no real or potential conflicts of interest to declare. This work was supported by a grant-in-aid for early-career scientists from the Japan Society for the Promotion of Science (grant number 18K16362).

AUTHOR CONTRIBUTIONS

Conception and design: S. Kikuchi and T.F.; development of methodology: T. Ogawa, S. Kikuchi, and T.F.; acquisition of data: T. Ogawa, M.T., E.M., Y.U., and S. Kikuchi; analysis and interpretation of data: T. Ogawa, S. Kikuchi, H.T., K.N., and T.F.; writing, review, and/or revision of the manuscript: T. Ogawa and S. Kikuchi; administrative, technical, or material support: Y.U.; study supervision: S. Kikuchi, S. Kuroda, K.N., T. Ohara, and S. Kagawa, and T.F.

DECLARATION OF INTERESTS

Yasuo Urata is the president and CEO of Oncolys BioPharma, the manufacturer of OBP-702. Hiroshi Tazawa and Toshiyoshi Fujiwara are consultants for Oncolys BioPharma. The other authors have no real or potential conflicts of interest to declare.

REFERENCES

1. Chau, I., Norman, A.R., Cunningham, D., Waters, J.S., Oates, J., and Ross, P.J. (2004). Multivariate prognostic factor analysis in locally advanced and metastatic esophago-gastric cancer–pooled analysis from three multicenter, randomized, controlled trials using individual patient data. J. Clin. Oncol. *22*, 2395–2403. https://doi.org/10.1200/jco.2004.08.154.
2. Kalluri, R. (2016). The biology and function of fibroblasts in cancer. Nat. Rev. Cancer *16*, 582–598. https://doi.org/10.1038/nrc.2016.73.
3. Quail, D.F., and Joyce, J.A. (2013). Microenvironmental regulation of tumor progression and metastasis. Nat. Med. *19*, 1423–1437. https://doi.org/10.1038/nm.3394.
4. Yasuda, T., Koiwa, M., Yonemura, A., Miyake, K., Kariya, R., Kubota, S., Yokomizo-Nakano, T., Yasuda-Yoshihara, N., Uchihara, T., Itoyama, R., et al. (2021). Inflammation-driven senescence-associated secretory phenotype in cancer-associated fibroblasts enhances peritoneal dissemination. Cell Rep. *34*, 108779. https://doi.org/10.1016/j.celrep.2021.108779.
5. Öhlund, D., Elyada, E., and Tuveson, D. (2014). Fibroblast heterogeneity in the cancer wound. J Exp Med. *211*, 1503–1523.
6. Procopio, M.G., Laszlo, C., Al Labban, D., Kim, D.E., Bordignon, P., Jo, S.H., Goruppi, S., Menietti, E., Ostano, P., Ala, U., et al. (2015). Combined CSL and *p53* downregulation promotes cancer-associated fibroblast activation. Nat. Cell Biol. *17*, 1193–1204. https://doi.org/10.1038/ncb3228.
7. Schmid, J.O., Dong, M., Haubeiss, S., Friedel, G., Bode, S., Grabner, A., Ott, G., Murdter, T.E., Oren, M., Aulitzky, W.E., and van der Kuip, H. (2012). Cancer cells cue the *p53* response of cancer-associated fibroblasts to cisplatin. Cancer Res. *72*, 5824–5832. https://doi.org/10.1158/0008-5472.can-12-1201.
8. Arandkar, S., Furth, N., Elisha, Y., Nataraj, N.B., van der Kuip, H., Yarden, Y., Aulitzky, W., Ulitsky, I., Geiger, B., and Oren, M. (2018). Altered *p53* functionality in cancer-associated fibroblasts contributes to their cancer-supporting features. Proc. Natl. Acad. Sci. U S A. *115*, 6410–6415. https://doi.org/10.1073/pnas.1719076115.
9. Bieging, K.T., Mello, S.S., and Attardi, L.D. (2014). Unravelling mechanisms of *p53*-mediated tumour suppression. Nat. Rev. Cancer *14*, 359–370. https://doi.org/10.1038/nrc3711.
10. Muller, P.A.J., and Vousden, K.H. (2013). *p53* mutations in cancer. Nat. Cell Biol. *15*, 2–8. https://doi.org/10.1038/ncb2641.
11. Fang, B., and Roth, J.A. (2003). Tumor-suppressing gene therapy. Cancer Biol. Ther. *2*, 114–120. https://doi.org/10.4161/cbt.210.
12. Fujiwara, T., Tanaka, N., Kanazawa, S., Ohtani, S., Saijo, Y., Nukiwa, T., Yoshimura, K., Sato, T., Eto, Y., Chada, S., et al. (2006). Multicenter phase I study of repeated

intratumoral delivery of adenoviral *p53* in patients with advanced non-small-cell lung cancer. J. Clin. Oncol. *24*, 1689–1699. https://doi.org/10.1200/jco.2005.03.4116.

13. Nemunaitis, J., Clayman, G., Agarwala, S.S., Hrushesky, W., Wells, J.R., Moore, C., Hamm, J., Yoo, G., Baselga, J., Murphy, B.A., et al. (2009). Biomarkers predict *p53* gene therapy efficacy in recurrent squamous cell carcinoma of the head and neck. Clin. Cancer Res. *15*, 7719–7725. https://doi.org/10.1158/1078-0432.ccr-09-1044.

14. Pagliaro, L.C., Keyhani, A., Williams, D., Woods, D., Liu, B., Perrotte, P., Slaton, J.W., Merritt, J.A., Grossman, H.B., and Dinney, C.P. (2003). Repeated intravesical instillations of an adenoviral vector in patients with locally advanced bladder cancer: a phase I study of *p53* gene therapy. J. Clin. Oncol. *21*, 2247–2253. https://doi.org/10.1200/jco.2003.09.138.

15. Kawashima, T., Kagawa, S., Kobayashi, N., Shirakiya, Y., Umeoka, T., Teraishi, F., Taki, M., Kyo, S., Tanaka, N., and Fujiwara, T. (2004). Telomerase-specific replication-selective virotherapy for human cancer. Clin. Cancer Res. *10*, 285–292. https://doi.org/10.1158/1078-0432.ccr-1075-3.

16. Hashimoto, Y., Watanabe, Y., Shirakiya, Y., Uno, F., Kagawa, S., Kawamura, H., Nagai, K., Tanaka, N., Kumon, H., Urata, Y., and Fujiwara, T. (2008). Establishment of biological and pharmacokinetic assays of telomerase-specific replication-selective adenovirus. Cancer Sci. *99*, 385–390. https://doi.org/10.1111/j.1349-7006.2007.00665.x.

17. Sasaki, T., Tazawa, H., Hasei, J., Kunisada, T., Yoshida, A., Hashimoto, Y., Yano, S., Yoshida, R., Uno, F., Kagawa, S., et al. (2011). Preclinical evaluation of telomerase specific oncolytic virotherapy for human bone and soft tissue sarcomas. Clin. Cancer Res. *17*, 1828–1838. https://doi.org/10.1158/1078-0432.ccr-10-2066.

18. Ishikawa, W., Kikuchi, S., Ogawa, T., Tabuchi, M., Tazawa, H., Kuroda, S., Noma, K., Nishizaki, M., Kagawa, S., Urata, Y., and Fujiwara, T. (2020). Boosting replication and penetration of oncolytic adenovirus by paclitaxel eradicate peritoneal metastasis of gastric cancer. Mol. Ther. Oncolytics *18*, 262–271. https://doi.org/10.1016/j.omto.2020.06.021.

19. Nemunaitis, J., Tong, A.W., Nemunaitis, M., Senzer, N., Phadke, A.P., Bedell, C., Adams, N., Zhang, Y.A., Maples, P.B., Chen, S., et al. (2010). A phase I study of telomerase-specific replication competent oncolytic adenovirus (telomelysin) for various solid tumors. Mol. Ther. *18*, 429–434. https://doi.org/10.1038/mt.2009.262.

20. Shirakawa, Y., Tazawa, H., Tanabe, S., Kanaya, N., Noma, K., Koujima, T., Kashima, H., Kato, T., Kuroda, S., Kikuchi, S., et al. (2021). Phase I dose-escalation study of endoscopic intratumoral injection of OBP-301 (Telomelysin) with radiotherapy in oesophageal cancer patients unfit for standard treatments. Eur. J. Cancer *153*, 98–108. https://doi.org/10.1016/j.ejca.2021.04.043.

21. Hasei, J., Sasaki, T., Tazawa, H., Osaki, S., Yamakawa, Y., Kunisada, T., Yoshida, A., Hashimoto, Y., Onishi, T., Uno, F., et al. (2013). Dual programmed cell death pathways induced by *p53* transactivation overcome resistance to oncolytic adenovirus in human osteosarcoma cells. Mol. Cancer Ther. *12*, 314–325. https://doi.org/10.1158/1535-7163.mct-12-0869.

22. Koujima, T., Tazawa, H., Ieda, T., Araki, H., Fushimi, T., Shoji, R., Kuroda, S., Kikuchi, S., Yoshida, R., Umeda, Y., et al. (2020). Oncolytic virus-mediated targeting of the ERK signaling pathway inhibits invasive propensity in human pancreatic cancer. Mol. Ther. Oncolytics *17*, 107–117. https://doi.org/10.1016/j.omto.2020.03.016.

23. Tanimoto, T., Tazawa, H., Ieda, T., Nouso, H., Tani, M., Oyama, T., Urata, Y., Kagawa, S., Noda, T., and Fujiwara, T. (2020). Elimination of MYCN-amplified neuroblastoma cells by telomerase-targeted oncolytic virus via MYCN suppression. Mol. Ther. Oncolytics *18*, 14–23. https://doi.org/10.1016/j.omto.2020.05.015.

24. Hasovits, C., and Clarke, S. (2012). Pharmacokinetics and pharmacodynamics of intraperitoneal cancer chemotherapeutics. Clin. Pharmacokinet. *51*, 203–224. https://doi.org/10.2165/11598890-000000000-00000.

25. Markman, M., Bundy, B.N., Alberts, D.S., Fowler, J.M., Clark-Pearson, D.L., Carson, L.F., Wadler, S., and Sickel, J. (2001). Phase III trial of standard-dose intravenous cisplatin plus paclitaxel versus moderately high-dose carboplatin followed by intravenous paclitaxel and intra- peritoneal cisplatin in small-volume stage III ovarian carcinoma: an intergroup study of the Gynecologic Oncology Group, Southwestern Oncology Group, and Eastern Cooperative Oncology Group. J. Clin. Oncol. *19*, 1001–1007. https://doi.org/10.1200/jco.2001.19.4.1001.

26. Armstrong, D.K., Bundy, B., Wenzel, L., Huang, H.Q., Baergen, R., Lele, S., Copeland, L.J., Walker, J.L., and Burger, R.A.; Gynecologic Oncology Group (2006). Intraperitoneal cisplatin and paclitaxel in ovarian cancer. N. Engl. J. Med. *354*, 34–43. https://doi.org/10.1056/nejmoa052985.

27. Trimble, E.L., and Christian, M.C. (2006). Intraperitoneal chemotherapy for women with advanced epithelial ovarian carcinoma. Gynecol. Oncol. *100*, 3–4. https://doi.org/10.1016/j.ygyno.2005.12.006.

28. Ishigami, H., Fujiwara, Y., Fukushima, R., Nashimoto, A., Yabusaki, H., Imano, M., Imamoto, H., Kodera, Y., Uenosono, Y., Amagai, K., et al. (2018). Phase III trial comparing intraperitoneal and intravenous paclitaxel plus S-1 versus cisplatin plus S-1 in patients with gastric cancer with peritoneal metastasis: PHOENIX-GC trial. J. Clin. Oncol. *36*, 1922–1929. https://doi.org/10.1200/jco.2018.77.8613.

29. Fujiwara, T., Kagawa, S., and Tazawa, H. (2012). Synergistic interaction of telomerase-specific oncolytic virotherapy and chemotherapeutic agents for human cancer. Curr. Pharm. Biotechnol. *13*, 1809–1816. https://doi.org/10.2174/138920112800958887.

30. Fujiwara, T., Kagawa, S., Kishimoto, H., Endo, Y., Hioki, M., Ikeda, Y., Sakai, R., Urata, Y., Tanaka, N., and Fujiwara, T. (2006). Enhanced antitumor efficacy of telomerase-selective oncolytic adenoviral agent OBP-401 with docetaxel: preclinical evaluation of chemovirotherapy. Int. J. Cancer *119*, 432–440. https://doi.org/10.1002/ijc.21846.

31. Paget, S. (1989). The distribution of secondary growths in cancer of the breast. 1889. Cancer Metastasis Rev. 8, 98–101.

32. Yashiro, M., Chung, Y.S., Nishimura, S., Inoue, T., and Sowa, M. (1996). Fibrosis in the peritoneum induced by scirrhous gastric cancer cells may act as "soil" for peritoneal dissemination. Cancer *77*, 1668–1675. https://doi.org/10.1002/(sici)1097-0142(19960415)77:8+<1668::aid-cncr13>3.0.co;2-w.

33. Sakamoto, S., Kagawa, S., Kuwada, K., Ito, A., Kajioka, H., Kakiuchi, Y., Watanabe, M., Kagawa, T., Yoshida, R., Kikuchi, S., et al. (2019). Intraperitoneal cancer-immune microenvironment promotes peritoneal dissemination of gastric cancer. Oncoimmunology *8*, e1671760. https://doi.org/10.1080/2162402x.2019.1671760.

34. Ebbing, E.A., van der Zalm, A.P., Steins, A., Creemers, A., Hermsen, S., Rentenaar, R., Klein, M., Waasdorp, C., Hooijer, G.K.J., Meijer, S.L., et al. (2019). Stromal-derived interleukin 6 drives epithelial-to-mesenchymal transition and therapy resistance in esophageal adenocarcinoma. Proc. Natl. Acad. Sci. U S A. *116*, 2237–2242. https://doi.org/10.1073/pnas.1820459116.

35. Karakasheva, T.A., Lin, E.W., Tang, Q., Qiao, E., Waldron, T.J., Soni, M., Klein-Szanto, A.J., Sahu, V., Basu, D., Ohashi, S., et al. (2018). IL-6 mediates cross-talk between tumor cells and activated fibroblasts in the tumor microenvironment. Cancer Res. *78*, 4957–4970. https://doi.org/10.1158/0008-5472.can-17-2268.

36. Kato, T., Noma, K., Ohara, T., Kashima, H., Katsura, Y., Sato, H., Komoto, S., Katsube, R., Ninomiya, T., Tazawa, H., et al. (2018). Cancer-associated fibroblasts affect intratumoral CD8(+) and FoxP3(+) T cells via IL6 in the tumor microenvironment. Clin. Cancer Res. *24*, 4820–4833. https://doi.org/10.1158/1078-0432.ccr-18-0205.

37. Johnson, D.E., O'Keefe, R.A., and Grandis, J.R. (2018). Targeting the IL-6/JAK/STAT3 signalling axis in cancer. Nat. Rev. Clin. Oncol. *15*, 234–248. https://doi.org/10.1038/nrclinonc.2018.8.

38. Ham, I.H., Oh, H.J., Jin, H., Bae, C.A., Jeon, S.M., Choi, K.S., Son, S.Y., Han, S.U., Brekken, R.A., Lee, D., and Hur, H. (2019). Targeting interleukin-6 as a strategy to overcome stroma-induced resistance to chemotherapy in gastric cancer. Mol. Cancer *18*, 68. https://doi.org/10.1186/s12943-019-0972-8.

39. Addadi, Y., Moskovits, N., Granot, D., Lozano, G., Carmi, Y., Apte, R.N., Neeman, M., and Oren, M. (2010). *p53* status in stromal fibroblasts modulates tumor growth in an SDF1-dependent manner. Cancer Res. *70*, 9650–9658. https://doi.org/10.1158/0008-5472.can-10-1146.

40. Moskovits, N., Kalinkovich, A., Bar, J., Lapidot, T., and Oren, M. (2006). *p53* attenuates cancer cell migration and invasion through repression of SDF-1/CXCL12 expression in stromal fibroblasts. Cancer Res. *66*, 10671–10676. https://doi.org/10.1158/0008-5472.can-06-2323.

41. Bar, J., Feniger-Barish, R., Lukashchuk, N., Shaham, H., Moskovits, N., Goldfinger, N., Simansky, D., Perlman, M., Papa, M., Yosepovich, A., et al. (2009). Cancer cells suppress *p53* in adjacent fibroblasts. Oncogene *28*, 933–936. https://doi.org/10.1038/onc.2008.445.

42. Takakura, M., Nakamura, M., Kyo, S., Hashimoto, M., Mori, N., Ikoma, T., Mizumoto, Y., Fujiwara, T., Urata, Y., and Inoue, M. (2010). Intraperitoneal

administration of telomerase-specific oncolytic adenovirus sensitizes ovarian cancer cells to cisplatin and affects survival in a xenograft model with peritoneal dissemination. Cancer Gene Ther. *17*, 11–19. https://doi.org/10.1038/cgt.2009.44.

43. Liu, D., Kojima, T., Ouchi, M., Kuroda, S., Watanabe, Y., Hashimoto, Y., Onimatsu, H., Urata, Y., and Fujiwara, T. (2009). Preclinical evaluation of synergistic effect of telomerase-specific oncolytic virotherapy and gemcitabine for human lung cancer. Mol. Cancer Ther. *8*, 980–987. https://doi.org/10.1158/1535-7163.mct-08-0901.

44. Osaki, S., Tazawa, H., Hasei, J., Yamakawa, Y., Omori, T., Sugiu, K., Komatsubara, T., Fujiwara, T., Sasaki, T., Kunisada, T., et al. (2016). Ablation of MCL1 expression by virally induced microRNA-29 reverses chemoresistance in human osteosarcomas. Sci. Rep. *6*, 28953. https://doi.org/10.1038/srep28953.

45. Galanis, E., Hartmann, L.C., Cliby, W.A., Long, H.J., Peethambaram, P.P., Barrette, B.A., Kaur, J.S., Haluska, P.J., Jr., Aderca, I., Zollman, P.J., et al. (2010). Phase I trial of intraperitoneal administration of an oncolytic measles virus strain engineered to express carcinoembryonic antigen for recurrent ovarian cancer. Cancer Res. *70*, 875–882. https://doi.org/10.1158/0008-5472.can-09-2762.

46. Galanis, E., Atherton, P.J., Maurer, M.J., Knutson, K.L., Dowdy, S.C., Cliby, W.A., Haluska, P., Jr., Long, H.J., Oberg, A., Aderca, I., et al. (2015). Oncolytic measles virus expressing the sodium iodide symporter to treat drug-resistant ovarian cancer. Cancer Res. *75*, 22–30. https://doi.org/10.1158/0008-5472.can-14-2533.

47. Noma, K., Smalley, K.S., Lioni, M., Naomoto, Y., Tanaka, N., El-Deiry, W., King, A.J., Nakagawa, H., and Herlyn, M. (2008). The essential role of fibroblasts in esophageal squamous cell carcinoma-induced angiogenesis. Gastroenterology *134*, 1981–1993. https://doi.org/10.1053/j.gastro.2008.02.061.

48. Chou, T.C. (2006). Theoretical basis, experimental design, and computerized simulation of synergism and antagonism in drug combination studies. Pharmacol. Rev. *58*, 621–681. https://doi.org/10.1124/pr.58.3.10.

OMTO, Volume *25*

Supplemental information

Modulation of *p53* expression in cancer-associated fibroblasts prevents peritoneal metastasis of gastric cancer

Toshihiro Ogawa, Satoru Kikuchi, Motoyasu Tabuchi, Ema Mitsui, Yuta Une, Hiroshi Tazawa, Shinji Kuroda, Kazuhiro Noma, Toshiaki Ohara, Shunsuke Kagawa, Yasuo Urata, and Toshiyoshi Fujiwara

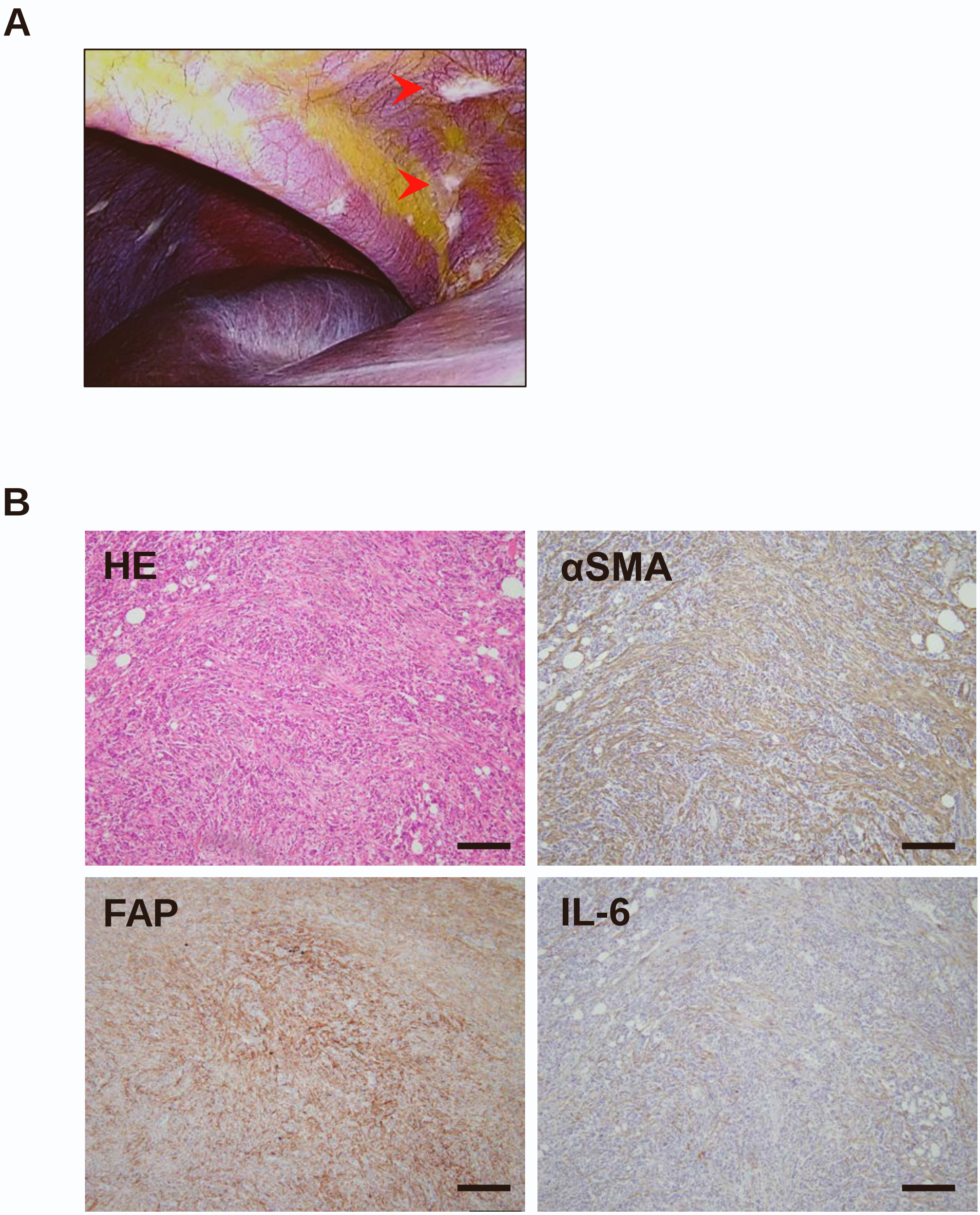

Supplementary Fig. S1

Immunohistochemical analysis of peritoneal metastasis of GC. **A,** Laparoscopic view of peritoneal metastasis of GC. White nodules indicates peritoneal metastasis (arrowheads). **B,** Representative microscopic images of peritoneal metastasis stained by HE and IHC staining for αSMA, FAP and IL-6. Scale bar, 200µm.

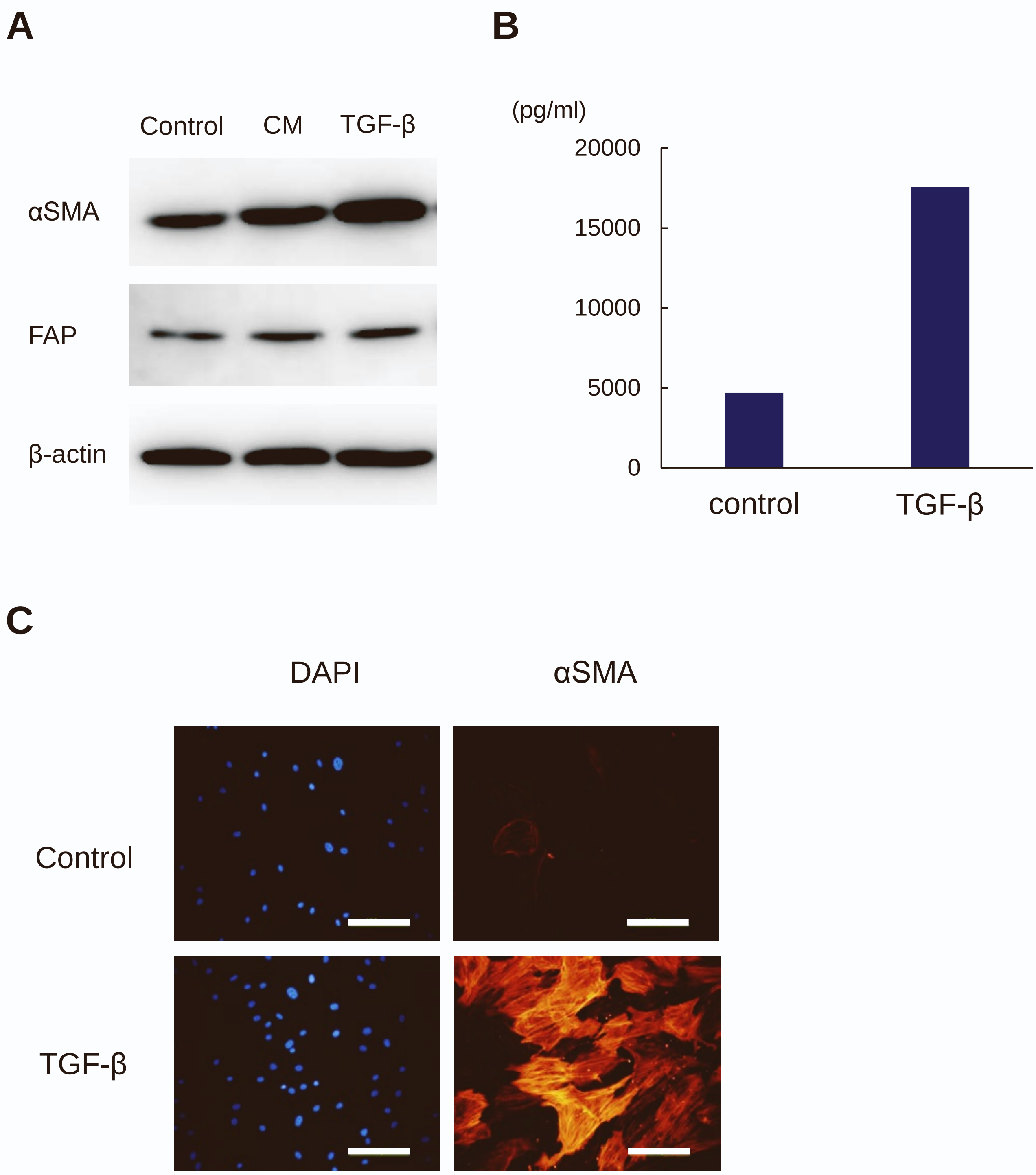

Supplementary Fig. S2

NGF activated by CM from MKN-7 or TGF-β showed CAFs-like properties. **A,** Western blotting analysis of NGF and activated NGF by CM from MKN-7 or TGF-β. β-actin was used as a loading control.. **B,** The amount of IL-6 secretion in NGF and activated NGF by TGF-β. **C,** Immunofluorescence analysis of NGF and activated NGF by TGF-β. Blue color indicates the nucleus, orange color indicates αSMA expression. Scale bar, 200μm.

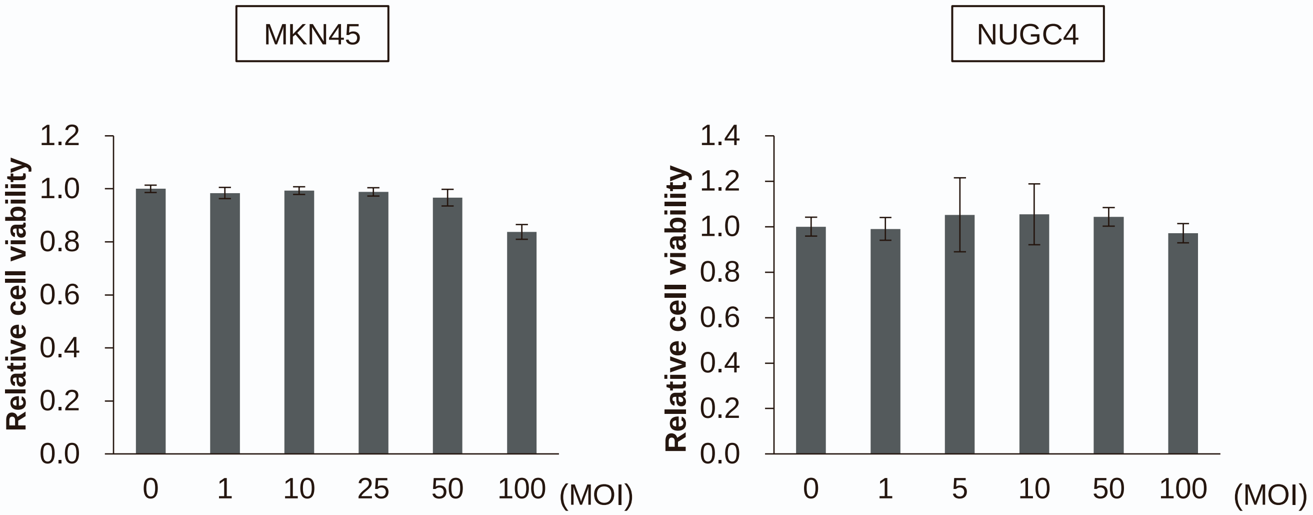

Supplementary Fig. S3

MKN45 and NUGC4 cells were resistant to OBP-301. Cells were infected with OBP-301 at the indicated MOIs for 3 days. Cell viability was quantified using the XTT assay. The cell viability of a mock-treated group was considered 1.0, and the relative cell viability was calculated. Data are expressed as the mean ± SD (n = 5).

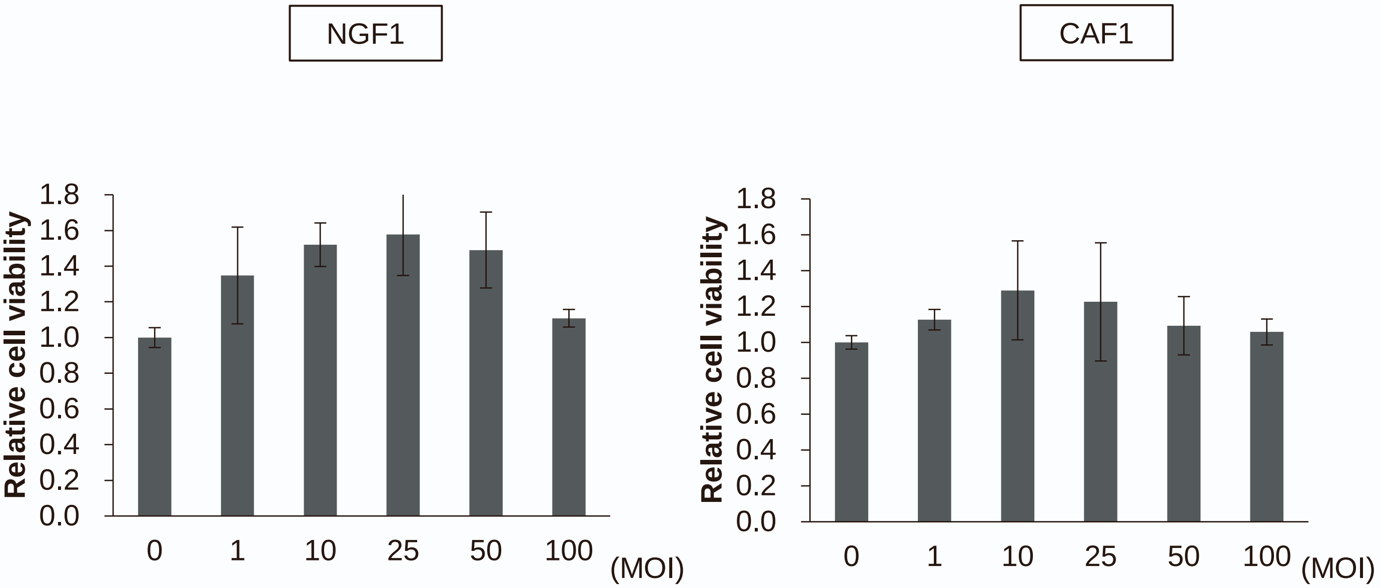

Supplementary FigS4

OBP-301 has no cytotoxicity to NGF and CAF. NGF1 and CAF1 were infected with OBP-301 at the indicated MOIs for 3 days. Cell viability was quantified using the XTT assay. The cell viability of a mock-treated group was considered 1.0, and the relative cell viability was calculated. Data are expressed as the mean ± SD (n = 5).

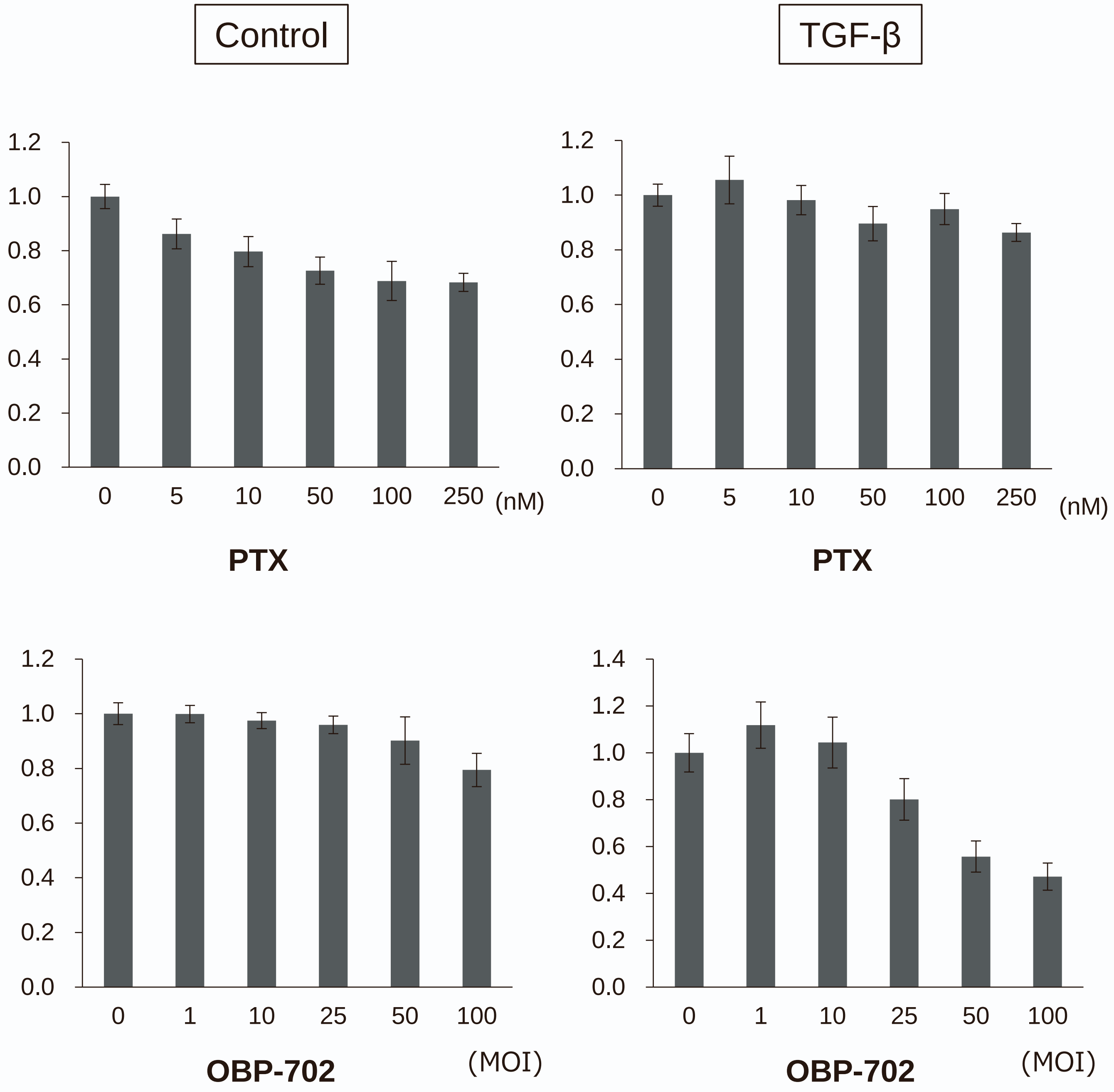

Supplementary FigS5

Activated NGF by TGF-β became to be OBP-702 sensitive and PTX resistant. NGF2 and activated NGF2 by TGF-β were infected with OBP-702 at the indicated MOIs for 3 days. Cells were treated with PTX at the indicated doses for 24 h. Cell viability was quantified using the XTT assay. The cell viability of a mock-treated group was considered 1.0, and the relative cell viability was calculated. Data are expressed as the mean ± SD (n = 5).

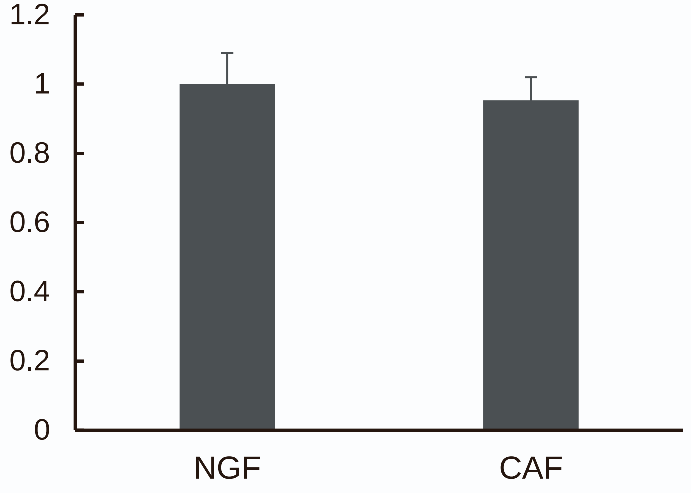

Supplementary FigS6

hTERT expression between NGF and CAF. Quantitative real-time PCR assay was performed to quantify the amount of hTERT copy. The copy number of hTERT is defined as the hTERT/ GAPDH ratio relative to that of the sample NGF (NGF = 1). Data are shown as means ± SD.

A

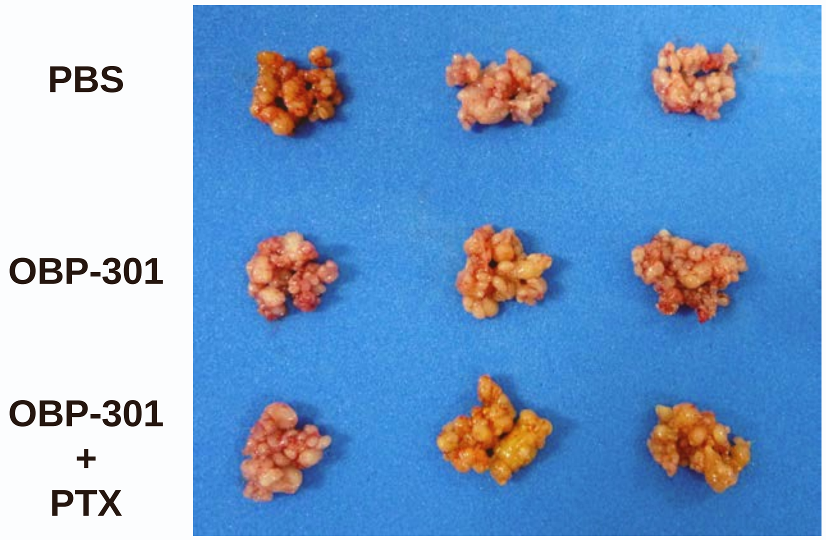

B

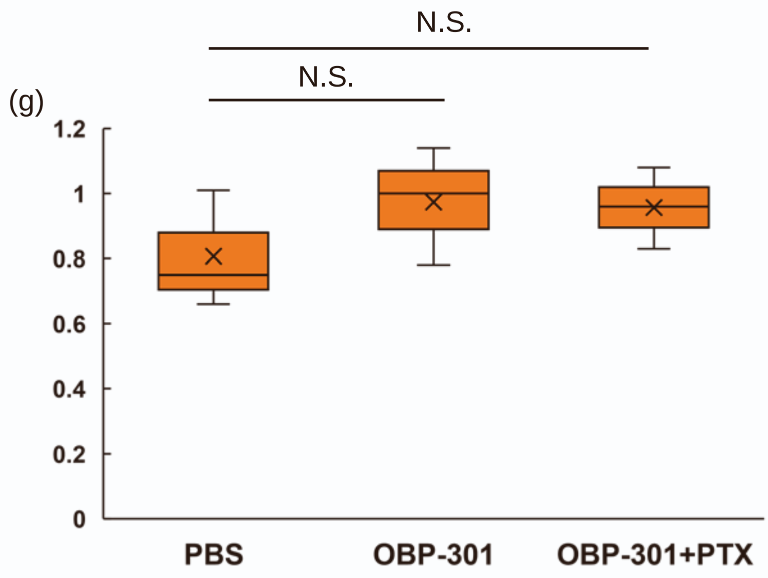

Supplementary FigS7
Intraperitoneal administration of OBP-301 in combination with PTX did not suppress tumor growth of MKN45-Luc and CAFs. **A,** Macroscopic images of resected all tumor nodules in abdominal cavity after treatment. B, Total tumor volume weight in abdominal cavity after treatment. Data are expressed as mean ± SD (n = 3). Statistical significance was defined as $p < 0.05$.
